# RpS19a depletion alters the hematopoietic translatome to drive tissue overgrowth

**DOI:** 10.1016/j.isci.2026.117515

**Published:** 2026-09-15

**Authors:** Naomi C. Mitchell, Arjun Chahal, Tanya Javaid, Katrina Woodward, Ralf B. Schittenhelm, Cheng Huang, Julie Lorent, Jiwon Lee, Melanie Rug, Amee J. George, Ross D. Hannan, Ola Larsson, Anthony W. Purcell, Eduardo Eyras, Nikolay Shirokikh, Teresa T. Bonello, Leonie M. Quinn, Olga Zaytseva

**Affiliations:** 1 Division of Genome Science and Cancer, The John Curtin School of Medical Research, The Shine-Dalgarno Centre for RNA Innovation, The Australian National University, Canberra, ACT 2601, Australia; 2 Department of Biochemistry and Molecular Biology, Biomedicine Discovery Institute, Monash University, Clayton, VIC 3800, Australia; 3 Monash Proteomics & Metabolomics Platform, Biomedicine Discovery Institute, Monash University, Clayton, VIC 3800, Australia; 4 Department of Oncology-Pathology, Science for Life Laboratory, Karolinska Institutet, 171 77 Stockholm, Sweden; 5 Centre for Advanced Microscopy, The Australian National University, Canberra, ACT 2601, Australia; 6 The Australian Centre for RNA Therapeutics in Cancer, School of Human Sciences, The University of Western Australia, Perth, WA 6009, Australia

**Keywords:** *Drosophila*, NEP1, RpS19a, blood, growth control, lymph gland, protein translation, ribosomal protein, ribosomopathy

## Abstract

Despite ribosomal protein loss correlating with increased tumor predisposition, direct mechanisms for the counterintuitive oncogenic effect of ribosomal protein depletion have not been reported. We used genetic models to investigate ribosomal protein S19a (RpS19a) depletion in the *Drosophila* blood organ, the lymph gland. Intriguingly, we demonstrate that RpS19a depletion directly drives excess proliferation and tissue overgrowth. Proteomic and differential translation analysis of RpS19a-depleted cells revealed altered stoichiometry of translation initiation factors and increased association of ribosomes with mRNAs encoding growth-promoting proteins. Furthermore, ribosomes in RpS19a-depleted cells are associated with mRNA encoding dNep1, a putative ribosomal RNA methyltransferase. Although uncharacterized in *Drosophila*, NEP1 is implicated in ribosomal RNA methylation and ribosome stability in yeast and humans, and its mutations underpin the ribosomopathy Bowen-Conradi syndrome. The RpS19a knockdown phenotype is suppressed by dNep1 co-depletion, altogether suggesting that dNep1 enables assembly of pro-proliferative ribosomes essential for blood lineage overgrowth driven by ribosomal protein loss.

## Highlights

• Ribosomal protein depletion in the *Drosophila* lymph gland drives tissue overgrowth
• Loss of RpS19a increases ribosomal translation of growth-promoting factors
• RpS19a loss phenotype is suppressed by dNep1 rRNA methyltransferase co-depletion
• Altered translation is a mechanism for oncogenic effects of ribosomal protein loss

## Graphical abstract

**Figure:**
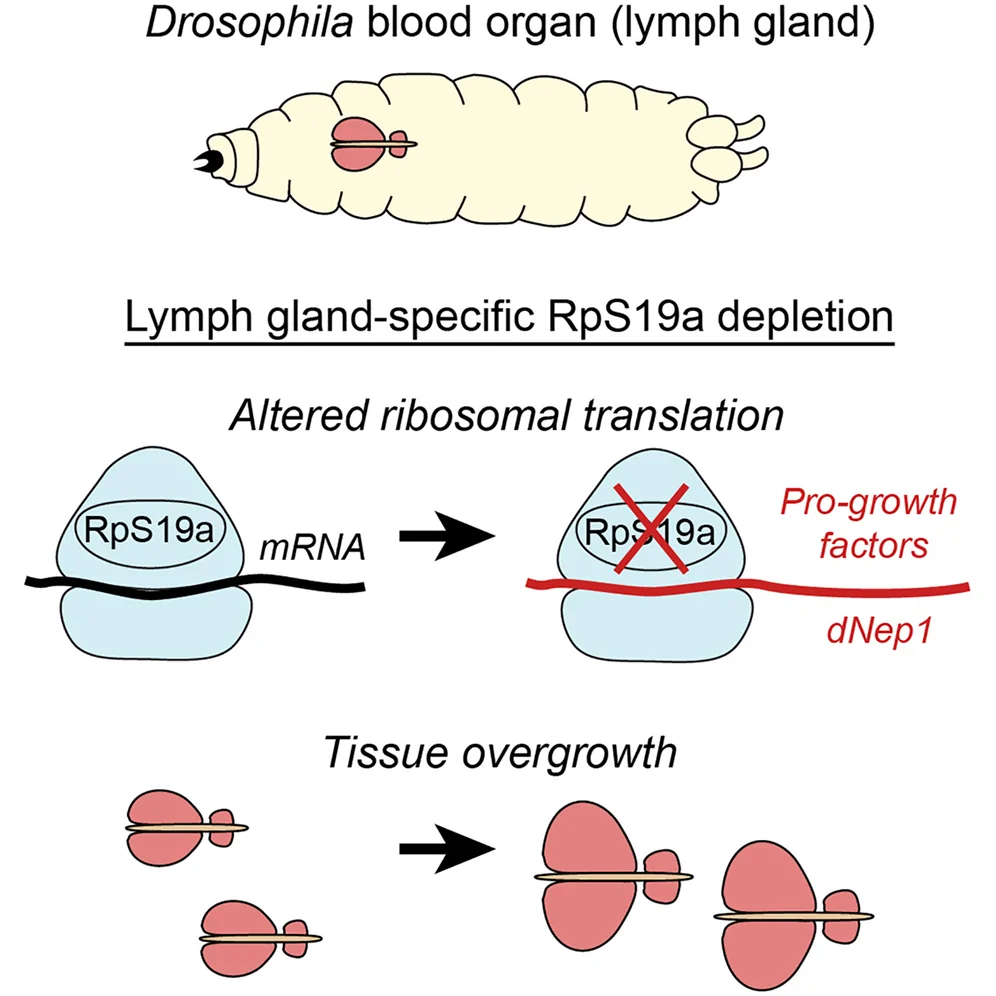

## Teaser

Biochemistry; Biological sciences; Protein; Protein physics

## Introduction

The eukaryotic (80S) ribosome, comprised of a small 40S and large 60S subunit assembled from 4 ribosomal RNA (rRNA) molecules and ∼80 ribosomal proteins (RPs), is essential for mRNA translation and generation of the intricate array of proteins necessary for life. Thus, dysregulation of ribosome biogenesis correlates with disease. On the one hand, ribosomes are hyper-abundant in cancer cells, while on the other, deficiency underlies ribosomopathies: congenital disorders with diverse phenotypes, predominantly characterized by hypo-proliferation-related symptoms (e.g., stunted growth and anemia).^1^^,^^2^^,^^3^ Less well defined are the paradoxical hyper-proliferation phenotypes and significantly elevated cancer risk observed in individuals with ribosomopathies^4^^,^^5^^,^^6^^,^^7^^,^^8^^,^^9^ and, more broadly, oncogenic roles suggested by the somatic hemizygosity of RPs observed in 43% of all cancers.^10^

Individuals diagnosed with the ribosomopathy Diamond-Blackfan anemia (DBA) usually present with hematopoietic stem and progenitor cell (HSPC) loss, erythroid depletion, bone marrow failure, red cell aplasia, and anemia^2^^,^^3^ but are also significantly more likely to develop leukemia^4^^,^^5^ and a range of solid cancers.^6^^,^^7^^,^^8^ The monoallelic loss of the 40S RP *RPS19* (eS19), which occurs most commonly in individuals with DBA (accounting for ∼25% of all diagnosed cases),^2^^,^^3^^,^^11^ leads to HSPC depletion. This occurs, at least in part, as a result of nucleolar surveillance pathway (NSP) activation,^12^ which drives release of free RPs that sequester the mouse double minute 2 homolog (MDM2, E3 ubiquitin-protein ligase), thus stabilizing p53 to promote cell cycle delay and/or apoptosis.^12^^,^^13^ Based on studies in DBA mouse models and patient samples, impaired translation of differentiation factors is also implicated in promoting erythroid depletion.^14^ Nevertheless, mechanisms for cancer predisposition and evolution of malignancies in DBA patients are less clear.^15^ For instance, while forward genetic mutagenesis screens in zebrafish identified haploinsufficiency for 11 RPs, including *RpS19*, in malignant nerve sheath tumors,^16^ the mechanisms underlying tumorigenesis remain obscure.

In *Drosophila*, tissue overgrowth observed in heterozygous mutants for certain RPs^17^^,^^18^ occurs via tissue-extrinsic mechanisms. Specifically, RP depletion impairs growth of the prothoracic hormone-secreting gland to delay animals in the larval feeding stage and, thus, indirectly increase body size.^18^^,^^19^ These observations are consistent with an essential function for RPs in promoting proliferative growth and are unlikely to explain why decreased ribosome function predisposes patients to cancer.^4^^,^^5^ The question, therefore, remains—why are DBA patients, with decreased erythroid progenitors, 28 times more likely to develop leukemia?^4^

We, therefore, modeled the most frequent mutation in DBA patients, i.e., depletion of *RPS19*,^2^^,^^3^^,^^11^ via knockdown (KD) of the *Drosophila* ortholog, RpS19a, in the hematopoietic compartment (lymph gland). We observed hyper-proliferation of blood cells and significant lymph gland overgrowth, providing a system to understand hematopoietic lineage expansion associated with RpS19a KD. Analysis of the stoichiometry of actively translating ribosomes (polysomes) from RpS19a KD blood lineage-derived S2 cells revealed that although overall levels of RpS19a were reduced in polysomes, the remaining protein continued to associate with polysomes, and we also observed altered stoichiometry of translation initiation factors (eIFs).

Moreover, RpS19a KD resulted in significant dysregulation of the translatome, including increased translation of highly conserved *Drosophila* nucleolar-expressed protein (*dNep1*, annotated as CG3527). Although uncharacterized in *Drosophila*, yeast Nep1 has been implicated in 40S assembly, enabling both 18S rRNA methylation and RPS19 incorporation into pre-ribosomes.^20^^,^^21^^,^^22^ The critical role for human NEP1 (encoded by the *EMG1* gene) in ribosome function is highlighted by the ribosomopathy Bowen-Conradi syndrome (BSC), where *NEP1* mutations lead to severe growth failure and infantile death.^23^ In addition to increased association with polysomes *ex vivo* (i.e., in RpS19a KD S2 cells), quantitative mass spectrometry revealed increased abundance of the NEP1 protein *in vivo*, i.e., in RpS19a KD lymph glands. Finally, the observation that dNep1 co-KD suppressed the RpS19a KD phenotype suggests increased dNep1 is required for lymph gland overgrowth. Together, our findings provide a model whereby altered translation can promote overproliferation, thus providing a direct mechanism for cancer predisposition in RPS19 mutant DBA patients.

## Results

### RpS19a KD drives proliferative overgrowth and depletes hematopoietic progenitors

**Figure 1:**
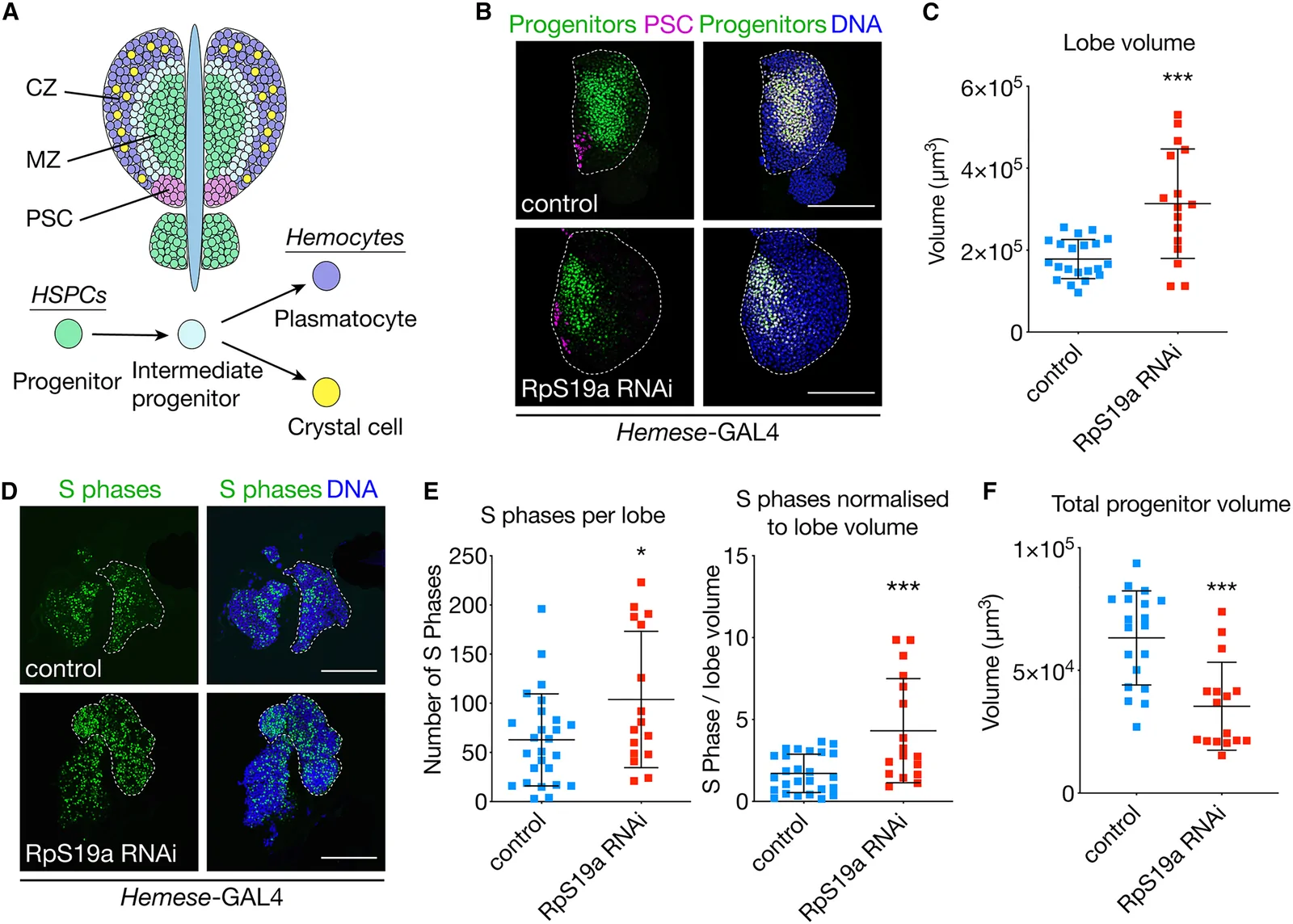
Lymph gland-specific RpS19a KD drives tissue overgrowth associated with progenitor depletion and differentiation defects (A) Schematic of a *Drosophila* larval lymph gland. 1° lobe showing cortical zone (CZ), medullary zone (MZ), and posterior signaling center (PSC). Progenitors transition to intermediate progenitors, which differentiate into plasmatocytes and crystal cells. (B) Confocal imaging for *Hemese*-*GAL4*/+ control and *Hemese*-*GAL4*/RpS19a RNAi lymph glands. 1° lobe outlined. Scale bars, 100 μm. (C) Volume of 1° lobe (*p* = 0.0001, Student’s *t* test, *n* > 16 biological replicates from >3 independent experiment repeats). (D) Confocal image of 5-ethynyl-2'-deoxyuridine (EdU)-positive cells. Scale bars, 100 μm. (E) Quantification of total S phases per 1° lobe (∗*p* = 0.0271, Student's *t* test, *n* > 16 biological replicates from >3 independent experiemtal repeats) and S phases normalized to lobe volume (∗∗∗∗*p* = 0.0004, Student’s *t* test, *n* > 16 biological replicates from >3 independent experiment repeats). (F) Total volume of *dome*-GFP stem and progenitor cells (*p* = 0.0001, Student’s *t* test, *n* > 16 biological replicates from >3 independent experiment repeats). Error bars represent mean and SD. See also Figures S1–S4.

Development of the primary blood repository in *Drosophila*, the lymph gland, is regulated by evolutionarily conserved modulators of hematopoietic stem cell fate,^24^^,^^25^ with the bulk of the blood lineage generated by the primary lymph gland lobes (Figure 1A).^26^^,^^27^ Although ubiquitous KD of RpS19a (using validated *UAS*-RpS19a RNAi hairpins, Figure S1A) impaired animal growth (Figure S1B), KD specifically in the lymph gland (driven by *Hemese*-GAL4^28^) resulted in tissue overgrowth (Figures 1B and 1C). Importantly, we also observed lymph gland overgrowth using a second, independent *UAS*-RpS19a RNAi (line 2, Figures S1C–S1E). KD of *RpS24*, the homolog of *RPS24* (eS24), which is also commonly mutated in DBA,^29^ significantly increased lymph gland volume and depleted progenitors (Figure S2). Thus, the phenotype is not specific to RpS19a KD, with depletion of a second 40S RP resulting in hematopoietic compartment overgrowth. Moreover, the RpS19a KD overgrowth phenotype was associated with significantly increased S phase progression (Figures 1D and 1E) and mitosis (Figure S3), both overall and when normalized to primary lobe volume.

Given the role of RPs as essential components of the ribosome and requirement for cell and tissue growth,^30^ the ectopic proliferation and tissue overgrowth associated with RpS19a KD was unexpected. Notably, lymph gland overgrowth occurred despite a reduction in the HSPC compartment (Figure 1F). Indeed, RP depletion is predicted to decrease ribosome assembly and activate cell-cycle arrest and/or apoptosis via the nucleolar stress response (NSP)^31^; the mechanism underlying erythroid lineage depletion and anemia in ribosomopathies.^13^^,^^32^ In *Drosophila,* activation of the NSP is mediated by Jun N-terminal kinase (JNK)-dependent cell death.^33^^,^^34^^,^^35^ While caspase activation was increased in RpS19a-depleted lymph glands (Figures S4A and S4B), JNK signaling (*puc-GFP*, an indirect measure of NSP) was decreased, suggesting RpS19a KD-associated death is not dependent on JNK activation (Figures S4C and S4D).

### RpS19a KD drives hematopoietic differentiation defects

**Figure 2:**
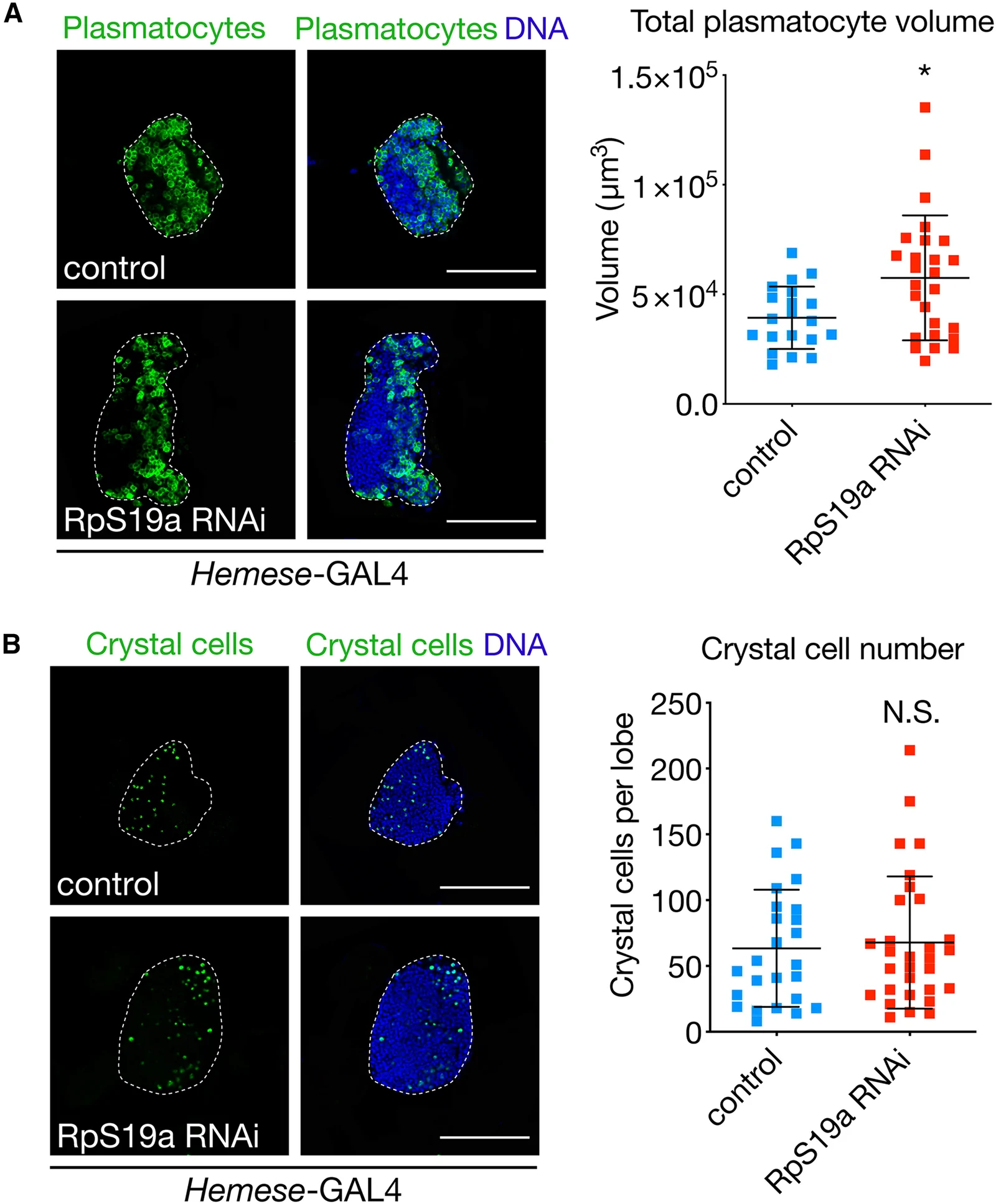
Lymph gland RpS19a KD drives expansion of plasmatocytes but not crystal cells (A) Total volume of NimC1-positive plasmatocytes (*p* = 0.0122, Student’s t test, *n* > 20 biological replicates from >3 independent experiment repeats). Scale bars, 100 μm. (B) Hindsight positive crystal cells (N.S. = not significant, Student’s *t* test, *n* > 25 biological replicates from >3 independent experiment repeats). Scale bars, 100 μm. Error bars represent mean and SD. See also Figures S5–S7.

In addition to tissue overgrowth and HSPC depletion (Figure 1), lymph gland-specific RpS19a KD resulted in differentiation defects. The posterior signaling centre (PSC) of the lymph gland controls HSPC renewal,^36^^,^^37^ promoting differentiation of stem cells into intermediate progenitors, which differentiate further to generate plasmatocytes for phagocytosis of microbial pathogens and dead cells,^38^^,^^39^ or melanin-producing crystal cells required for wound healing^40^ (Figure 1A). RpS19a KD increased plasmatocytes (Figure 2A) but did not significantly alter crystal cell number (Figure 2B). The moderate increase in plasmatocytes, compared to the overall increase in lobe volume, suggests expansion of additional populations, such as intermediate progenitors for which we lack definitive markers,^27^^,^^41^ contributes to overgrowth. Together, the phenotypic analysis demonstrates RpS19a KD alters hematopoietic cell fate to result in blood compartment overgrowth, via significant expansion of differentiated blood lineages at the expense of the HSPC population. To determine the molecular basis of lymph gland overgrowth, we next profiled the RpS19a KD transcriptome and proteome.

### RpS19a depletion alters the lymph gland transcriptome and proteome

The RpS19a transcriptome, determined via differential expression analysis of RNA sequencing (RNA-seq) data for RpS19a KD compared with control (DESeq2 R package analysis^42^ of raw gene-level counts, false discovery rate [FDR] < 0.01), included 1,255 differentially expressed genes (447 mRNAs increased and 808 decreased, Figure S5A; Data S1). Consistent with lymph gland overproliferation, ontology analysis using the clusterProfiler R package^43^ identified enrichment for mitotic cell cycle genes in the RpS19a KD lymph glands, including upregulation of positive cell cycle regulators PCNA, CycA, and 14-3-3ε, while downregulated transcripts were enriched for differentiation and developmental gene clusters (Figures S5B and S5C; Data S2). In line with an expansion of intermediate progenitors, we observed an increase in abundance of the *CG18547* transcript (Data S1), a reported marker for the GST-rich intermediate progenitor subpopulation.^41^ Furthermore, in support of the lineage-specific differentiation defects observed for RpS19a KD (i.e., plasmatocyte expansion and unaltered crystal cells, Figure 2), abundance of the plasmatocyte receptor *NimC1*^44^ was increased, while crystal cell markers *lz* and *PPO1* were unaltered (Data S1).

To characterize the RpS19a KD proteome, we used untargeted quantitative proteomics, which identified 2,860 proteins (Data S3) with abundance of 192 proteins significantly altered between RpS19a KD and control lymph glands (136 increased and 56 decreased, FDR < 0.05, Figure S6A). We observed significant differences for proteins implicated in ribosome biogenesis and translation (Figure S6B; Data S4) and, of note, increased abundance of numerous translation initiation factors (eIF2A, eIF3B, eIF3H, eIF3K, eIF4A, eIF4G1, and eIF6), translation elongation factors (eEF1-beta, eEF1-delta, and eEF2), 40S RPs (RpS4, RpS12, RpS15Aa, and RpS18), and 60S RPs (RpL7-like, RpL9, RpL10Ab, RpL23, RpL23A, RpL24, RpL30, RpL31, RpL35, and RpLP0) (Figure S6C). As increased abundance of ribosomal components and translation factors may occur, at least in part, as a consequence of overproliferation associated with RpS19a KD (i.e., due to the global increase in translation required for cell cycle progression^45^), to determine whether proteome dysregulation was also associated with altered translation, we next analyzed the composition of translational complexes and the translatome.

### RpS19a KD increases translation initiation factors eIF4A and eIF5 on polysomes

**Figure 3:**
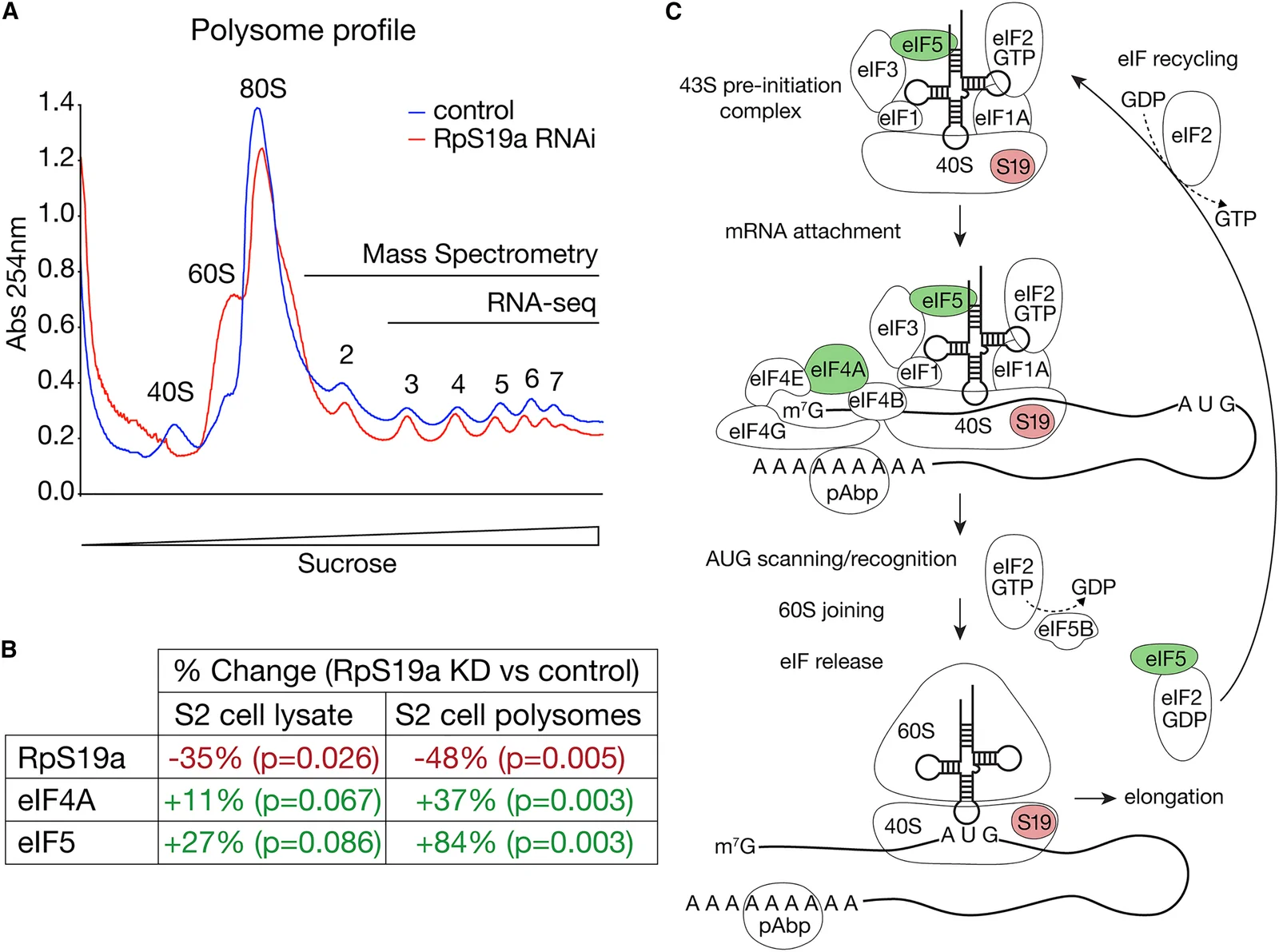
RpS19a KD alters ribosomal assembly and translation machinery (A) Polysome profiles of the control and RpS19a KD S2 cells. Fractions collected for RNA-seq and mass spectrometry are indicated. (B) Significantly altered protein abundance of translation factors in RpS19 KD polysomes indicates increased eIF4A and eIF5. FDR cutoff of 0.05 was applied to determine statistical significance. (C) Increased (green) and decreased (red) components in the context of their function in translation. See also Figure S8 and Data S5.

The overproliferation of RpS19a KD hemocytes (Figure 1E; Figure S3C) is paradoxical, as proliferation requires intact ribosome function, yet RP depletion is expected to impair 80S ribosome assembly.^46^ In accordance with continued ribosome assembly, electron microscopy detected ribosomes on the endoplasmic reticulum of RpS19a-KD lymph gland cells and further revealed increased mitochondria (Figure S7), consistent with the heightened metabolic pathway activity expected given the observed proliferative overgrowth (Figure 1). Due to the heterogeneous nature of lymph gland cell populations, in order to further investigate ribosome assembly associated with RpS19a KD, we turned to blood lineage-derived *Drosophila* S2 cells. Ribosome profiling of RpS19a KD S2 cells (Figure S8) enabled isolation of poly-ribosomes (polysomes), assemblies of multiple ribosomes associated with actively translated mRNA, to reveal formation of 80S complexes and polysomes following RpS19a KD (Figure 3A), in accordance with continued ribosome assembly and translation, respectively. Thus, mature ribosomes formed despite the reduction in 40S and accumulation of 60S ribosomal subunits observed in RpS19a KD ribosome profiles.

Quantitative proteomic analysis of RpS19a KD S2 cells, compared with scrambled short hairpin RNA (shRNA) control, revealed a 35% decrease in RpS19a protein in cell lysates (Figure 3B and Data S5), a reduction comparable to that reported for individuals with DBA.^47^ Moreover, stoichiometric analysis of polysomes from S2 cells with RpS19a KD revealed decreased abundance of RpS19a, compared with scrambled shRNA control (by 48%, Figure 3B). We did not, however, observe increased abundance of the related RpS19b paralog (with 66% amino acid similarity^48^) on KD polysomes, i.e., in place of RpS19a (Data S5), an observation consistent with the low-level expression of RpS19b in non-germline tissues^49^ and failure to detect RpS19b peptides in larval lymph glands by mass spectrometry (Data S3). The decreased RpS19a stoichiometry in polysomes suggests, at the level of individual ribosomes, RpS19a incorporation does not always occur.

Although the abundance of most translation initiation factors, which interact with the 40S to control translational selectivity, was unchanged on RpS19a KD polysomes (Data S5), initiation factors eIF4A and eIF5 were both increased in RpS19a KD S2 cell lysates and on polysomes (Figures 3B and 3C). Given that the observed overall decrease in subunit 40S components, eIF4A and eIF5 may preferentially associate with the remaining initiating 40S subunits. As an ATP-dependent DEAD box RNA helicase, increased eIF4A on RpS19a KD polysomes has the potential to facilitate unwinding of complex 5′ untranslated regions (5′ UTRs) for certain mRNA species,^50^^,^^51^^,^^52^ and thus alter hematopoietic translational programs. Moreover, given that overexpression of human eIF5 promotes preferential translation of *ATF4* proto-oncogene mRNA,^53^ elevated eIF5 may also alter translation in RpS19a KD S2 cells. Thus, we next aimed to determine if RpS19a KD altered translational selection.

### RpS19a KD results in differential mRNA abundance on polysomes

**Figure 4:**
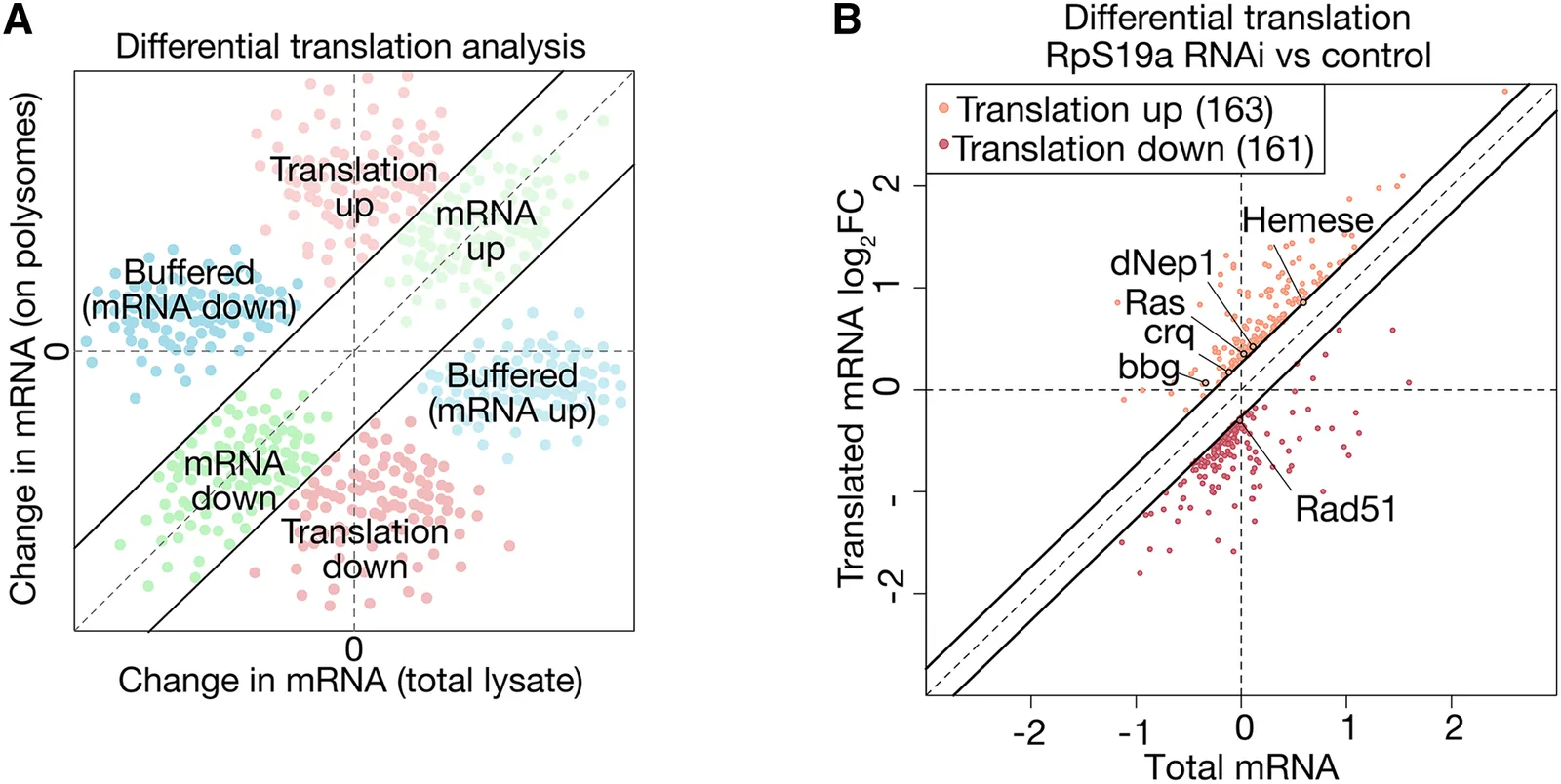
RpS19a KD results in differential translation in S2 cells (A) Anota2seq analysis comparing relative changes in total mRNA versus translated mRNA (associated with polysomes). Changes in association with polysomes without proportional total mRNA change are indicative of differential translation (red). (B) Anota2seq differential translation in RpS19 KD S2 cells compared to control (scrambled small interfering RNA [siRNA]). FDR <0.15 was used to determine statistical significance, *n* = 3 biological replicates. See also Data S6.

To determine whether RpS19a KD alters the translatome, we sequenced poly(A) selected total mRNA (input) and mRNA from polysome fractions and determined differential translation using anota2seq^54^^,^^55^ (Figure 4A). 324 mRNAs were unaltered in input but significantly changed on RpS19a KD polysomes compared with control (161 decreased and 163 increased), which encoded cell cycle regulators (e.g., CycJ), growth factors (FGF ortholog, Btl), oncogenes (e.g., Ras ortholog Ras 64B), and factors involved in blood lineage specification (e.g., Hemese, crq, and bbg) (Figure 4B and Data S6). Collectively, these data suggest RpS19a KD qualitatively alters ribosome composition and quantitatively alters selection of mRNA species for translation.

### RpS19a KD increases abundance of dNep1 mRNA on polysomes

To identify translational changes with potential *in vivo* significance, we intersected differentially translated mRNAs, determined by the translational profiling of RpS19a KD S2 cells previously (Data S6), with proteins significantly altered in RpS19a KD lymph glands (by mass spec) but unaltered at the mRNA level (by RNA-seq) (Data S1 and S3). Overlap between the datasets was limited to 3 genes, including *Canopy b*, *sesB*, and currently uncharacterized *CG3527*, the putative ortholog of nucleolar-expressed protein (NEP1). We predict the limited overlap is not only a result of differences between *in vivo* and *in vitro* systems but also the timing of KD (performed throughout lymph gland development using *Hemese*-GAL4, in contrast to transient KD in S2 cells). It is, therefore, likely that other factors, important for the RpS19a KD lymph gland phenotype, were not captured in this study.

**Figure 5:**
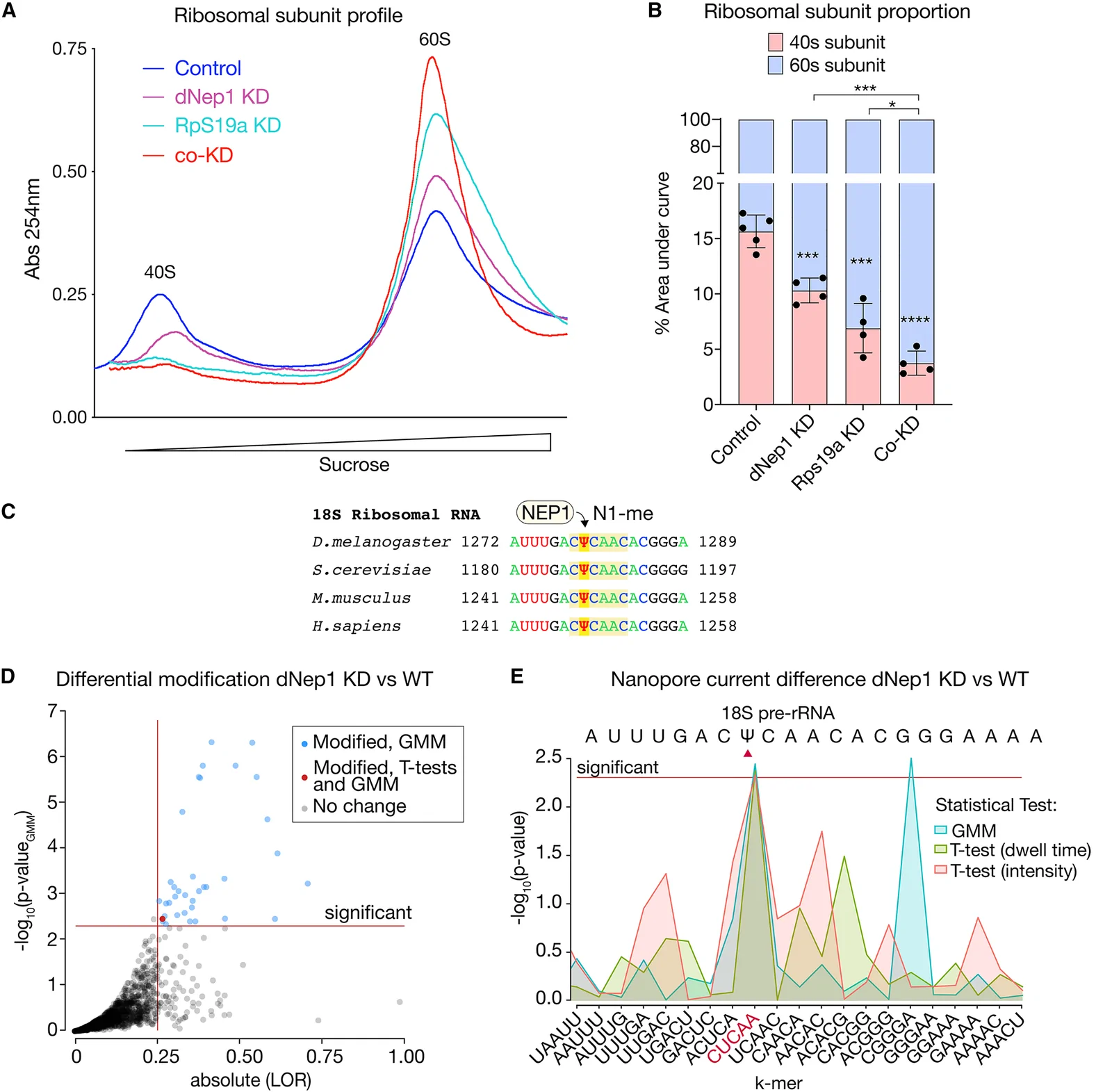
dNep1 is required for ribosome assembly and 18S rRNA modification (A) High-salt ribosome subunit profiles for S2 cells for control (untreated), dNep1 KD, RpS19a KD, or co-KD. (B) Quantification of area under curve corresponding to 40S and 60S subunit proportions (∗∗∗∗*p* < 0.0001, ∗∗∗*p* < 0.001, ∗*p* < 0.05; Student’s *t* test; error bars represent mean and SD, *n* = 3 biological replicates). (C) NEP1 consensus on 18S rRNA. (D) Analysis of direct RNA sequencing signals of 18S rRNA from dNep1 KD and wild-type (WT) S2 cells using Nanocompore. A Gaussian mixture model (GMM) bivariate statistical test considering both signal intensity and dwell time was used to generate a logistic regression log-odds ratio and *p* values. K-mers were considered differentially modified between samples if a significance threshold of *p* < 0.005 and an absolute log-odds ratio (LOR) exceeding 0.25 was achieved (blue). A single site has simultaneously reached *p* < 0.005 in the bivariate test and univariate *t* tests assessing either intensity or dwell time (red). (E) Plot of bivariate GMM and univariate *t* tests (dwell time, signal intensity). *p* values across *Drosophila* 18S rRNA reference (NCBI reference sequence: NR_133550.1) from dNep1 KD compared to WT S2 cells. K-mer overlapping position 1,279 of the hypermodified Ψ is the only position in which all statistical tests show high significance (*p* < 0.005). See also Figures S9 and S10.

Nevertheless, the NEP1 methyltransferase is essential for ribosome biogenesis^21^^,^^56^ and mutations associated with the ribosomopathy Bowen-Conradi syndrome.^23^^,^^57^^,^^58^ Yeast and human NEP1 localize to the nucleolus and are predicted to regulate 40S biogenesis, via 18S-rRNA precursor methylation and promotion of RPS19 incorporation.^56^^,^^59^^,^^60^ Ribosomal subunit profiling, following dNep1 (*Drosophila* Nep1) KD in S2 cells (Figure S9A), revealed decreased 40S formation (Figures 5A and 5B), consistent with previous yeast studies.^56^^,^^60^ Individual KD of RpS19a also significantly decreased 40S abundance, while dNep1 co-KD further significantly decreased 40S formation, compared with either dNep1 or RpS19a KD alone (Figure S9B; Figures 5A and 5B).

As the N1/1,248 residue in the defined 18S rRNA binding site for NEP1 (5′-CUCAAC-3′) is conserved between *Drosophila*, yeast, mice, and humans (Figure 5C), we tested whether dNep1 KD alters methylation of 18S rRNA using Oxford Nanopore single-molecule long-read sequencing to detect modified and unmodified nucleotides.^61^ To detect differential levels of methylation, 18S rRNA molecule signals were extracted from direct RNA-seq. Analysis using Nanocompore’s bivariate model of changes in both signal intensity and dwell time identified 36 k-mer positions with statistically significant differences between control and dNep1 KD (Figure 5D). Of these sites, only one k-mer (5′CUCAA3′) also reached the significance threshold in univariate *t* tests for signal intensity and dwell time, supporting an overall shift in the level of 18S rRNA methylation (Figure 5D). This single robustly altered k-mer in the 18S rRNA reference overlaps with the consensus motif of NEP1 methylation at position 1,279 (5′CUCAA3′) (Figure 5E), corresponding to the conserved mammalian site at position 1,248. Visualization of the signal/dwell time distributions and mean values confirmed an overall shift at position 1,279 for dNep1 KD compared with control, consistent with differential residue modification (Figure S9).

### dNep1 is required for lymph gland overgrowth driven by RpS19a KD

**Figure 6:**
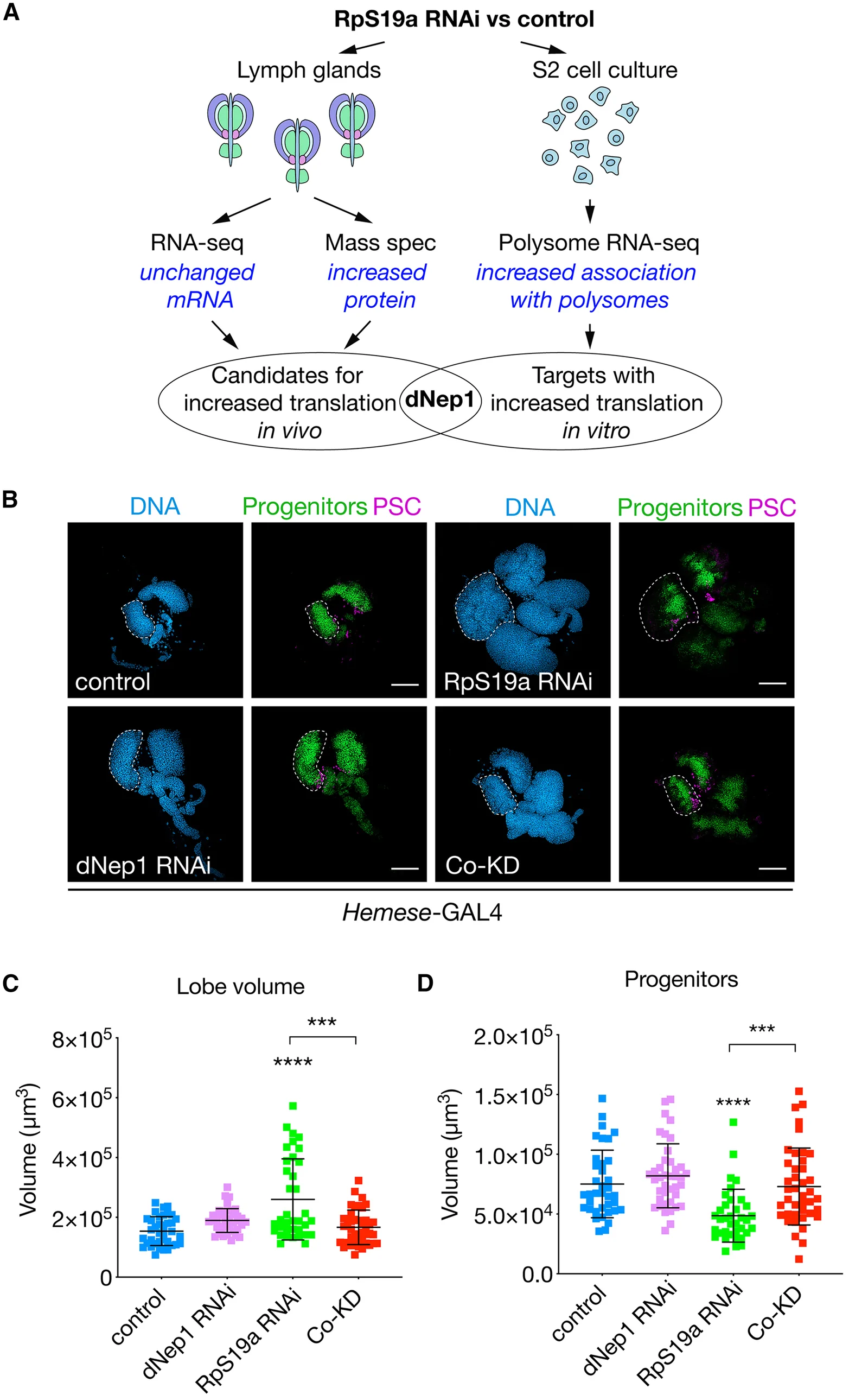
dNep1 is a differentially translated target following RpS19a KD *in vivo* and *in vitro*, required for lymph gland overgrowth (A) dNep1 is identified as both differentially translated in RpS19a KD S2 cells and increased at the protein level in RpS19a KD lymph glands. (B–D) Lymph gland primary lobes (outlined, genotypes marked) (B) with quantification of lobe volume (C) and progenitors (D). Scale bars, 100 μm (∗∗∗∗*p* < 0.0001, ∗∗∗*p* < 0.001 using Student’s *t* test, *n* > 35 biological replicates from >3 independent experiment repeats; error bars represent mean and SD). See also Figure S10.

**Figure 7:**
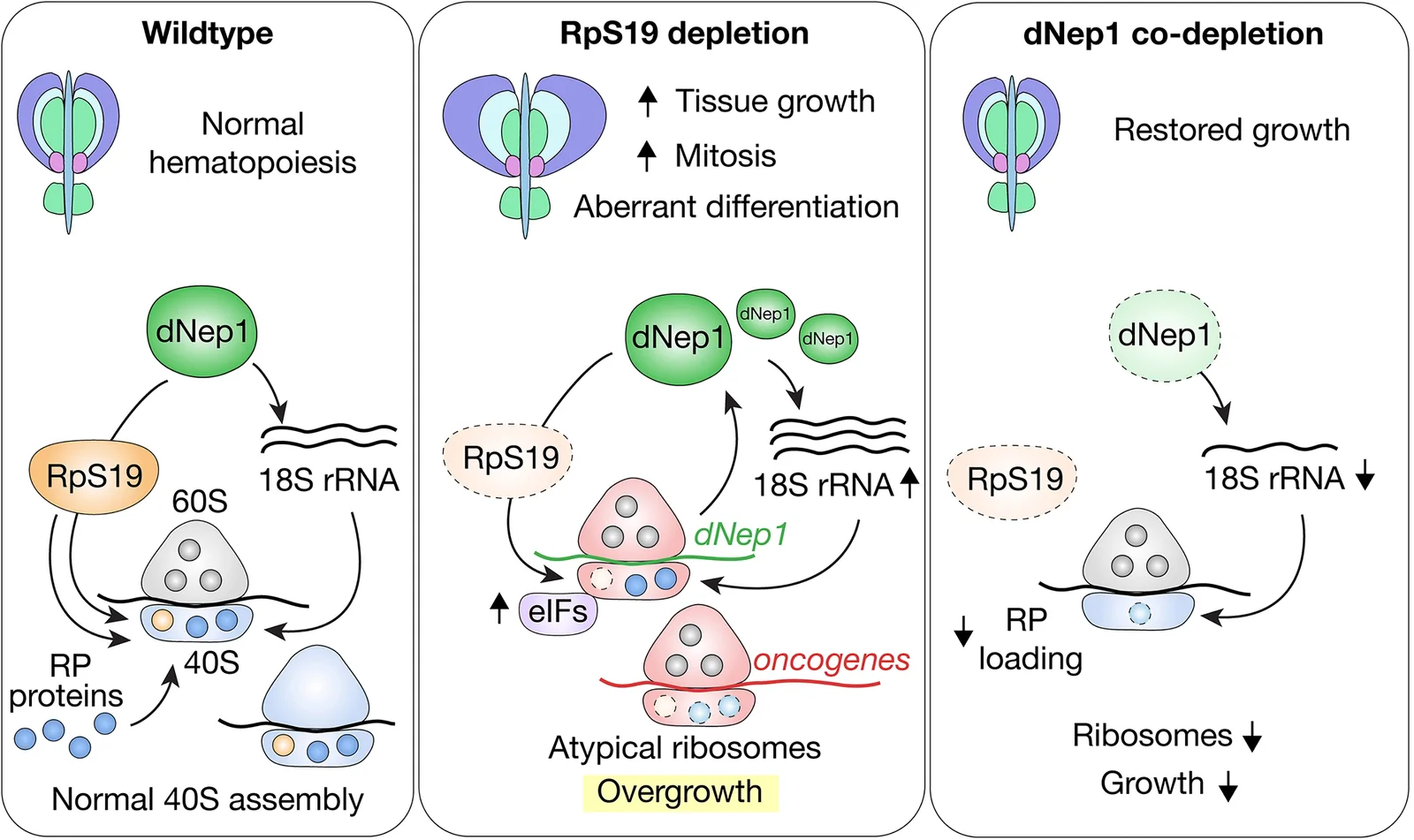
RpS19a depletion alters ribosome stoichiometry and translation to drive hematopoietic overgrowth Working model: RpS19a KD results in hematopoietic tissue overgrowth and defective differentiation due to formation of atypical ribosomes, which differentially associate with eIFs and mRNAs to increase translation of growth-promoting genes and the ribosome biogenesis factor dNep1. Co-depletion of dNep1 restores tissue growth by further depleting the pool of functional ribosomes.

As *dNep1* (*CG3527*) mRNA was significantly increased on RpS19a KD polysomes in S2 cells and dNep1 protein was elevated in RpS19a KD lymph glands (Figure 6A), we tested whether dNep1 KD (Figure S10C) modified the RpS19a KD phenotype. Indeed, consistent with increased dNep1 mediating the RpS19a KD phenotype, dNep1 co-KD not only suppressed lymph gland overgrowth but also restored stem/progenitor cells back to the control range (Figures 6B–6D). dNep1 methylation of 18S rRNA, the structural core of the 40S ribosomal subunit, is predicted to facilitate the 40S assembly and 80S stability essential for translational speed and accuracy.^20^^,^^21^^,^^22^ Given that mutations in yeast NEP1 result in accumulation of rRNA processing intermediates, indicative of defective rRNA processing,^59^ we measured levels of the rRNA internal transcribed spacer 1 (*ITS1*) for both dNep1- or RpS19a-depleted lymph glands. We did not, however, observe a significant change in *ITS1* RNA in dNep1 KD lymph glands (Figure S9D), suggesting altered 40S ribosome assembly (Figure 5B) may be due to defective rRNA processing downstream of *ITS1* cleavage. In contrast, *ITS1* was significantly increased by RpS19a KD and co-KD in lymph glands (Figure S9D), consistent with ribosome biogenesis defects observed for RpS19a KD (Figure 3B). We predict that in the RpS19a KD lymph glands, impaired ribosome biogenesis together with dNep1’s capacity to promote RpS19 incorporation^62^ facilitates formation of defective ribosomes, thus enabling proliferative translational programs (Figure 7).

## Discussion

RP depletion is usually associated with impaired cell and tissue growth^63^; consistent with this, ribosomopathy patients suffer life-threatening anemia^2^^,^^3^ but, surprisingly, also experience a higher incidence of acute myeloid leukemia and solid tumors.^4^^,^^5^^,^^6^^,^^7^^,^^8^ The observation that leukemia frequently evolves from aplastic anemia is the basis for William Dameshek’s now-famous riddle, first posed in a 1967 Blood editorial^64^ in context with the observed shift from hypo- to hyper-proliferation phenotypes in the blood lineage. Although a definitive answer remains elusive, the dominant hypothesis posits impaired proliferation (due to ribosome depletion in the case of DBA) imposes strong pressure on cell populations, resulting in selection of cells with compensatory mutations that rescue the impaired proliferation. For example, acquisition of a secondary mutation downstream of the NSP (e.g., the p53-axis), a secondary effect of ribosome dysfunction, might indirectly result in progression from hypo- to hyper-proliferation in ribosomopathy patients.^9^^,^^15^

Here, we provide the first evidence that ribosome dysfunction can directly drive hematopoietic lineage overproliferation and blood compartment overgrowth, suggesting secondary, indirect effects of RP depletion do not provide the full solution to Dameshek’s riddle. Rather, our findings indicate RpS19a KD disrupts stoichiometry of translating ribosomes and alters the hematopoietic translatome to drive proliferative growth. For example, the observation that mRNAs encoding oncogenes are increased while tumor suppressors are decreased on RpS19a KD polysomes indicates tumors in ribosomopathy patients might also arise via pro-proliferative translational programs. Thus, formation of defective ribosomes and associated differential translation would provide a direct mechanism for acquisition of the clonal advantage necessary to promote tumorigenesis.

RP recruitment studies in yeast suggest RPS19 incorporation occurs late in 40S formation,^65^^,^^66^ providing a rationale for the formation of defective ribosomes lacking RpS19a observed here being associated with altered translational selectivity and/or elongation. Indeed, under stress, yeast generate ribosomes lacking Rps26, which leads to diminished recognition of optimal −4G Kozak sequences, shifting translation toward suboptimal mRNAs.^67^^,^^68^ Thus, structurally unique ribosomes have the potential to change the translatome by enabling selective mRNA translation. Whether translational defects in mammalian RPS19 KD cells are a consequence of non-canonical ribosomes^69^^,^^70^ is currently unclear; however, the ribosomal heterogeneity and disruption to the translatome associated with RpS19a KD observed here suggest hematopoietic phenotypes might arise, at least in part, due to variability in ribosome composition. Our study further detected increased abundance of translational initiation factors (eIF4A and eIF5) in association with RpS19a KD polysomes. Previous studies suggest altered composition of these determinants of ribosome selectivity can result in modified translational fidelity, which is further proposed to contribute to oncogenesis.^71^^,^^72^ Indeed, 40S subunits from yeast and mammals can incorporate 5–10 times more eIF4A (i.e., compared with eIF2, eIF3, or eIF4B^73^^,^^74^) to result in increased helicase activity and more efficient translation of mRNAs with highly structured 5′ UTRs.^75^ In the case of eIF5, which is critical for formation of the 43S pre-initiation complex^76^^,^^77^ and 80S initiation complex,^78^^,^^79^ overexpression has been implicated in generation of “onco-ribosomes” that promote translation of *ATF4* proto-oncogene mRNA in human cell systems.^53^ RPS19 depletion has also been reported to decrease translational efficiency of transcripts essential for differentiation of murine and human erythroblasts in cell culture models^14^^,^^80^ and impair translation of shorter mRNA transcripts with less complex 5′ structures, which are generally less efficiently translated.^80^^,^^81^ As increased eIF5 and eIF4A, in conjunction with decreased RpS19a, are predicted to alter mRNA selection via distinctive mechanisms, further studies are required to determine the contribution of each of these changes to translational selectivity.

Ribosomal diversity in RpS19a-depleted cells may also arise as a consequence of differential rRNA modification.^82^ Pseudouridylation and methylation modulate architecture of the four major 18S rRNA domains,^83^ with formation of the 5′ domain enabling RP recruitment into the pre-40S complex to generate ribosomes.^84^ NEP1 controls 40S maturation via methylation of the conserved pseudouridine 1,248 (Ψ1248) in 18S rRNA.^21^ In yeast *NEP1* mutants, loss of Ψ1248 methylation results in 18S rRNA precursor accumulation,^21^^,^^56^ while *NEP1* mutation in humans causes Bowen-Conradi syndrome, a severe ribosomopathy associated with abnormal development and early childhood death.^23^^,^^57^^,^^58^ It remains to be determined whether increased translation of NEP1 and/or differential rRNA modification contributes to hematopoietic compartment overgrowth in *RPS19* mutant patients. Given the high conservation of NEP1 function from yeast to humans, we predict the pro-proliferative translatome arises, at least in part, as a result of overproduction of m1Ψ without change to m1acp3Ψ. Consistent with this prediction, knockout of the ribosome maturation factor TSR3, which performs amino-carboxy-propyl transfer on m1Ψ,^85^ induces expression of ribosome biogenesis genes to promote malignancy phenotypes in colorectal cancer cell lines.^86^ Our observation that the *Drosophila* ortholog of TSR3, CG4338, was not differentially translated following RpS19a KD suggests that an imbalance of these rRNA-modifying factors contributes to m1Ψ rRNA accumulation. We, therefore, predict that HSPC loss and tissue overgrowth driven by RpS19a KD in the hematopoietic compartment, which is dependent on elevated dNep1, is likely associated with an increase in 18S rRNA methylation and abundance of ribosomes with impaired translational selectivity.

Even though rRNA processing defects have been reported in hematopoietic cells from individuals with *RPS19* gene mutations, including a 3- to 4-fold increase in the ratio of 21S to 18S pre-rRNA,^87^^,^^88^ whether this is dependent on NEP1 and/or associated with ribosome heterogeneity remains unclear. However, yeast studies suggest Nep1 might facilitate Rps19 incorporation into the pre-40S by potentially altering rRNA structure to stabilize association between Rps19 and 18S rRNA.^62^ Thus, increased translation of dNep1 in the RpS19a KD background might enable methylation of 18S rRNA to increase availability of aberrant ribosomes and, thus, translational programs promoting proliferation. Conversely, the hyper-modification normally observed for rRNA m1acp3Ψ is lost in around 22 cancer types, including in half of all colon cancers, and is predicted to drive tumor proliferation as a result of instability of the ribosomal decoding site.^86^ The observed increase in translation of dNep1, which is predicted to enable both incorporation of RpS19a and 18S rRNA methylation, therefore has potential to stabilize defective ribosomes and promote the aberrant translation driving lymph gland overgrowth. Given NEP1 function in 18S methylation is not essential, at least in yeast,^20^ a future investigation to determine the contribution of dNep1 methyltransferase catalytic activity to the overgrowth phenotype is warranted.

Although mechanisms underlying tumorigenesis in ribosomopathy patients are inevitably complex, together, our observations suggest that RP depletion can qualitatively change ribosome composition to favor translation of mRNAs promoting proliferation. As ribosome biogenesis^89^ and NEP1^20^ are conserved throughout eukaryotic evolution, such alterations to ribosomes may not only hold the key to understanding ribosomopathies but may also shed light on increased malignancy associated with RP deficiency in more than 43% of all cancers, thus paving the way for new therapies targeting tumors in these patients.

### Limitations of the study

Here, we have developed a *Drosophila* genetic model for RPS19 loss in the hematopoietic compartment, with important future studies required to investigate the relevance of our findings to human disease. For example, to confirm whether NEP1 is involved in oncogenic progression in DBA, proteomic and direct rRNA analysis of tumors isolated from patients with DBA could be conducted. Furthermore, although DBA is associated with loss of a variety of RPs, our studies have focused primarily on the most common (RPS19).^2^^,^^3^^,^^11^ While we show that hematopoietic compartment expansion is not limited to RpS19a, it remains to be determined whether increased translation of dNep1 and/or differential rRNA modification might contribute to phenotypes associated with loss of additional RPs in the hematopoietic compartment. Further studies are also required to determine the contribution of increased eIF5 and eIF4A association to translational selectivity and/or hematopoietic compartment expansion. Likewise, investigating the contribution of dNep1 methyltransferase catalytic activity to the overgrowth phenotype will further reveal the underlying mechanisms.

## Resource availability

### Lead contact

Requests for further information and resources should be directed to and will be fulfilled by the lead contact, Olga Zaytseva (olga.zaytseva@anu.edu.au).

### Materials availability

This study did not generate new, unique reagents.

### Data and code availability

• RNA-seq data for lymph glands and S2 cells have been deposited at Gene Expression Omnibus (GEO) as GSE236312 and are publicly available as of the date of publication.
• Mass spectrometry proteomics data for lymph glands and S2 cells have been deposited at ProteomeXchange Consortium via the PRIDE partner repository as PXD043115 and are publicly available as of the date of publication.
• Oxford Nanopore direct RNA sequencing data for S2 cells have been deposited at the NCBI Sequence Read Archive (SRA) under BioProject PRJNA1476679 and are publicly available as of the date of publication.
• This paper does not report original code.
• Any additional information required to reanalyze the data reported in this paper is available from the lead contact upon request.

## Acknowledgments

The authors give thanks for the generous contribution of reagents: anti-NimC1 (gift from I. Ando) and domeless-GFP (gift from U. Banerjee). The authors acknowledge support from Microscopy Australia at the Center for Advanced Microscopy, Australian National University, a facility enabled by National Collaborative Research Infrastructure Strategy (NCRIS), the Biomolecular Resource Facility supported by NCRIS, and the 10.13039/501100000947Australian Cancer Research Foundation. This study used BPA-enabled (Bioplatforms Australia)/NCRIS-enabled infrastructure located at the Monash Proteomics and Metabolomics Platform. This work was funded by the 10.13039/501100000923Australian Research Council grant DP170100823 (L.M.Q. and R.D.H.), 10.13039/501100000923Australian Research Council grant DP220101352 (R.D.H.), 10.13039/501100000925National Health and Medical Research Council grant GNT1158732 (A.J.G.), Captain Courageous Foundation (A.J.G.), Maddie Riewoldt’s Vision Foundation (A.J.G.), 10.13039/501100004359Swedish Research Council (Vetenskapsrådet) grant 2020-01665 (O.L.), 10.13039/501100002794Swedish Cancer Society (Cancerfonden) grant 22 2186 (O.L.), Stockholm Cancer Society (Cancerfonden) grant 211222 (O.L.), and National Health and Medical Research Investigator Grant GNT1175388 (N.S.).

## Author contributions

Conceptualization, N.C.M., A.J.G., R.D.H., A.W.P., M.R., T.T.B., L.M.Q., and O.Z.; methodology, N.C.M., T.J., O.Z., O.L., R.B.S., and N.S.; formal analysis, N.C.M., A.C., C.H., J. Lorent, K.W., and O.Z.; investigation, N.C.M., A.C., T.J., J. Lee, K.W., and O.Z.; visualization, N.C.M., A.C., K.W., and O.Z.; resources, N.C.M. and N.S.; software, E.E.and O.L.; supervision, A.J.G., E. E., N.S., L.M.Q., and O.Z.; writing – original draft, A.C., L.M.Q., and O.Z.; writing – review and editing, N.C.M., R.B.S., T.T.B., N.S., L.M.Q., and O.Z.; funding acquisition, L.M.Q., R.D.H., A.J.G., O.L., and N.S.

## Declaration of interests

The authors declare no competing interests.

## STAR★Methods

### Key resources table

**Table:** 

REAGENT or RESOURCE	SOURCE	IDENTIFIER
**Antibodies**		

Mouse anti-Hindsight (1:50)	Developmental Studies Hybridoma Bank	1G9; RRID: AB_528278
Rabbit Cleaved Caspase 1 (Dcp-1) (1:100)	Cell Signaling Technology	9578; RRID: AB_2721060
Mouse NimC1 (1:100)	I. Ando	N/A
Mouse anti-Antp (1:50)	Developmental Studies Hybridoma Bank	8C11; RRID: AB_528083
Rabbit anti-pH3 (1:2000)	Millipore	06-570; RRID: AB_310177
anti-Rabbit 488 (1:1000)	Jackson ImmunoResearch Laboratories Inc	711-545-152; RRID: AB_2313584
anti-Rabbit 649 (1:1000)	Jackson ImmunoResearch Laboratories Inc	711-175-152; RRID: AB_2340607
anti-Mouse 488 (1:1000)	Jackson ImmunoResearch Laboratories Inc	715-545-151; RRID: AB_2341099
anti-Mouse 594 (1:1000)	Jackson ImmunoResearch Laboratories Inc	715-585-151; RRID: AB_2340855
anti-Mouse 649 (1:1000)	Jackson ImmunoResearch Laboratories Inc	715-175-151: RRID: AB_2340820

**Deposited data**		

RNA-seq	Gene Expression Omnibus	GSE236312
Mass Spectrometry	ProteomeXchange Consortium	PXD043115
Oxford Nanopore Technologies direct RNA-seq	NCBI SRA	BioProject PRJNA1476679

**Experimental models: Cell lines**		

*Drosophila melanogaster*: Schneider’s S2 cells	ThermoFisher	R69007

**Experimental models: Organisms/strains**		

*Drosophila melanogaster*: UAS-RpS19a RNAi 1	Vienna Drosophila Resource Center	v107188
*Drosophila melanogaster*: UAS-RpS19a RNAi 2	Bloomington Drosophila Stock Center	BL42774
*Drosophila melanogaster*: UAS-RpS24 RNAi	Vienna Drosophila Resource Center	v104676
*Drosophila melanogaster*: UAS-dNep1 RNAi	Vienna Drosophila Resource Center	v24762
*Drosophila melanogaster*: w1118	Bloomington Drosophila Stock Center	BL39831
*Drosophila melanogaster*: Hemese-GAL4	Bloomington Drosophila Stock Center	BL8699
*Drosophila melanogaster*: tubulin-GAL4	Bloomington Drosophila Stock Center	BL5138
*Drosophila melanogaster*: tubulin-GAL80ts	Bloomington Drosophila Stock Center	BL7019
*Drosophila melanogaster*: domeless-GFP	U. Banerjee	N/A
*Drosophila melanogaster*: puckered-GFP	Bloomington Drosophila Stock Center	BL64449

**Oligonucleotides**		

qPCR Actin F: 5′ GGCGCAGAGCAAGCGTGGTA 3′	This paper	N/A
qPCR Actin R: 5′ GGGTGCCACACGCAGCTCAT 3′	This paper	N/A
qPCR Tbp F: 5′ CAGGGGCAAAGAGTGAGGAC 3′	This paper	N/A
qPCR Tbp R: 5′ AGGAACTTTGCAGGGAAACC 3′	This paper	N/A
qPCR RpS19a F: 5′ GCTGGAGTCGGTTCGATCAC 3′	This paper	N/A
qPCR RpS19a R: 5′ GTGCTTCTCGACCAAACGGG 3′	This paper	N/A
qPCR dNep1 F: 5′ GAGACGGTGAAGGTGCACAA 3′	This paper	N/A
qPCR dNep1 R: 5′ AGCCGGGATCTCTTTGGTTTT 3′	This paper	N/A
qPCR ITS1 F: 5′ GGGCATACGTTGGCTAATGC 3′	This paper	N/A
qPCR ITS1 R: 5′ TGGTTGGTTATGGGGTTTGCT 3′	This paper	N/A
dsRNA RpS19a+T7 F 5′ TAATACGACTCACTATAGGGCGTCACAGTGAAGGATATT 3′	This paper	N/A
dsRNA RpS19a+T7 R 5′ TAATACGACTCACTATAGGTGGTTAGCAATACGGTCCAG 3′	This paper	N/A
dsRNA dNep1+T7 F 5′ TAATACGACTCACTATAGGACTCCTCACGTCGTCTGAT 3′	This paper	N/A
dsRNA dNep1+T7 R 5′ TAATACGACTCACTATAGGCCGCCGAAAGTGGATAGTT 3′	This paper	N/A

**Software and algorithms**		

Imaris	Bitplane	9.0.2
Tophat2	RRID:SCR_013035	2.1.1
HTSeq	https://doi.org/10.1093/bioinformatics/btu638	0.6.1
DESeq2	https://doi.org/10.1186/s13059-014-0550-8	1.10.1
clusterProfiler	https://doi.org/10.1089/omi.2011.0118	2.4.3
MaxQuant	https://doi.org/10.1038/nbt.1511	1.5.3.30 and 1.6.016
Perseus	https://doi.org/10.1038/nmeth.3901	1.5.4.0 and 1.6.0.7
HiSAT2	RRID:SCR_015530	2.1.0
featureCounts	RRID:SCR_012919	1.6.4
anota2seq	https://doi.org/10.1093/nar/gkz223	1.0.1
Guppy	RRID:SCR_023196	5.0.7
Minimap2	RRID:SCR_018550	2.24
samtools	RRID: SCR_002105	1.12
bedtools	RRID: SCR_006646	2.28.0
Nanopolish	RRID:SCR_016157	0.13.3
NanopolishComp	https://github.com/a-slide/NanopolishComp	0.6.2
Nanocompore	RRID:SCR_017985	1.0.4

### Experimental model and study participant details

#### Experimental model – *Drosophila melanogaster*

*Drosophila melanogaster* stocks were maintained on a standard molasses and semolina *Drosophila* medium. Genetic crosses were raised at 29°C except when performed in the background of temperature-sensitive GAL80 expression, where they were initially raised at 18°C followed by a shift to 29°C. Details of fly stocks used can be found in the key resources table.

### Method details

#### Lymph gland immunostaining

Larval heads from late 3^rd^ instars (120 h post egg laying at 29°C) were dissected in 1× Phosphate Buffered Saline (PBS) and fixed for 30 min in 4% w/v paraformaldehyde (PFA) diluted with either PBS or 0.4% v/v Triton X-100 in PBS (PBT) and blocked in 10% normal goat serum (NGS) in PBT at room temperature for 1 h, followed by incubation overnight at 4°C with primary antibody. Tissues were then incubated with the appropriate fluorophore-tagged secondary antibodies for a minimum of 1 h at room temperature, before incubation in DAPI solution for 15 min. Washes in between incubations were performed with PBT, except for the 2 final washes after DAPI incubation, which were performed with PBS. Larval tissues were subsequently stored in 80% glycerol in PBS. Details of antibodies used can be found in the key resources table.

#### Lymph gland EdU staining

EdU staining was performed using the Click-IT EdU Alexa Fluor 647 Imaging kit (cat no C10340). Larval heads were dissected and kept in 1,000 μL ice-cold PBS for no longer than 30 min before addition of 1 μL 1,000× EdU (Component A from kit). Larval heads were incubated on the nutator in EdU solution for 30 min and then fixed in 4% w/v PFA in PBS for 20 min. Tissues were then rinsed twice in PBT and washed for 15 min 3 times in PBT, followed by 1 h incubation in 10% NGS/PBT and incubation in the appropriate primary antibodies overnight at 4°C. Larval heads were then rinsed twice and washed for 15 min 3 times in PBT before incubation in the appropriate secondary antibody for a minimum of 1.5 h at room temperature. The Click-IT cocktail was then prepared according to the manufacturer’s protocol and tissues were incubated for 30 min, followed by a 15 min PBT wash and a 10 min incubation in DAPI/PBT. Tissues were then rinsed three times and washed for 15 min 3 times in PBT before storage in 80% glycerol.

#### Microscopy

Lymph glands were imaged with the Zeiss LSM800 confocal microscope using Zen Blue Software. z series were performed with 1 μm sections and fluorophores imaged using band-pass filters to remove cross-detection between channels.

#### Correlative Light electron microscopy

Correlative Light and Electron Microscopy (CLEM)^90^ was used to locate fluorescently tagged hemocytes in *Drosophila* lymph gland for ultrastructure imaging. Lymph glands were dissected from control and *Hemese*-GAL4 driven RpS19a KD with *UAS*-RFP co-expression and subjected to high pressure freezing (Leica EM ICE). The samples were then freeze substituted (Leica AFS) and embedded into HM20 resin to preserve fluorescence and ultrastructure. The resin blocks were sectioned at ∼90 nm thickness (Leica UC7 ultramicrotome) onto London finder grids to aid correlation between two different microscopes. For imaging, the grid was first observed using a Zeiss LSM 800 confocal microscope (63× lens; 1.4 NA) to identify RFP+ve hemocytes with RpS19a knockdown. Samples were subsequently post-stained with uranyl acetate and lead citrate to enhance contrast during Transmission Electron Microscopy (TEM) imaging on a JEOL JEM-F200 (200 kV). Zen-connect (ZEISS proprietary software) enabled revisiting relevant fluorescent cells in the TEM by using the coordinates of the finder grid and creating final correlative images.

#### qPCR

RNA was isolated from either 10 larval heads or 20 lymph glands using the Promega ReliaPrep RNA Tissue miniprep system (Z6110) following the manufacturer’s protocol and eluted in 15 μL nuclease-free water. RNA purity and integrity were assessed using an automated electrophoresis system (2200 TapeStation, Agilent Technologies). For each cDNA synthesis reaction, 5 μL of the eluted RNA was used. The GoScript Reverse Transcription System kit from Promega was used for cDNA synthesis, following the manufacturer’s protocol. Both Oligo(dT) and random hexamer primers were used for the reaction and the tubes were incubated at 25°C for 5 min followed by 42°C for 30 min and finally at 85°C for 5 min to terminate the reaction. qPCR was performed using SYBR Green PCR Master Mix (Thermo Fisher Scientific). Each qPCR reaction contained 0.1 μL of cDNA, 5 μL SYBR Master Mix and 0.2 μM of each forward/reverse gene-specific primer in a final volume of 10 μL. qPCR reactions (95°C for 2 min, followed by 40 cycles of: 90°C for 5 s and 60°C for 20 s) were run using the StepOnePlus PCR System and Sequence Detection Systems in 96-well plates (ThermoFisher Scientific). Amplicon specificity was verified by melt curve analysis. Average Ct values for two technical replicates were calculated for each sample. Fold change was determined using the 2-ΔΔCT method. Where possible, primers were designed to span exon-exon junctions to prevent amplification of genomic DNA. The melt curve profile of the amplified target was inspected to verify specificity. For the qPCR reference, multiple internal control genes were analyzed for stability and target gene expression was normalized to the mean of *Tbp* and *Actin*, selected for having high expression and little sample-to-sample variability after RP knockdown, as determined by RefFinder method.^91^ Details of primers used can be found in the key resources table.

#### Lymph gland RNA-seq

Lymph glands were collected from 3^rd^ instar larvae (5 days post egg laying at 29°C). Due to differences in tissue size, approximately 120 control lymph glands were collected for controls, while 40 were collected for *RpS19a Hemese*-GAL4-driven KD. Samples from each genotype were pooled to ensure at least 900 ng of total RNA was prepared per sample, performed in triplicate. RNA was extracted as for qPCR. RNA integrity was checked using a TapeStation (Agilent Technologies), confirming RIN values above 9 for all samples. Library preparation was carried out at the Biomolecular Resource Facility (ANU). RNA was prepared using the standard TruSeq Illumina stranded protocol, with Oligo(dT) beads used to enrich for poly(A)-positive mRNA and exclude other RNA. Samples were sequenced using the HiSeq2500 Illumina system, using 125 bp paired end read configuration.

#### Lymph gland mass spectrometry

Larval lymph glands were collected from 3rd instar larvae (5 days post egg laying at 29°C) and each condition was run in triplicate. Due to differences in size, approximately 275 lymph glands were pooled for each control replicate while 200 were pooled for the RpS19a KD replicates, to ensure a minimum final amount of 400 μg of total protein per sample. Lymph glands were collected in 50 mM Tris-HCl pH 8, 15 mM KCl, 25 mM MgCl_2_, 1× Protease Inhibitors (Thermo Fisher Scientific, cat 87785), centrifuged at high speed and pellet snap frozen in liquid N_2_. Lymph glands were lysed by sonication in the presence of 1% (w/v) sodium deoxycholate (SDC) and Tris-HCl pH 8.1. SDC was removed by precipitation with 100% water-saturated ethyl acetate. All proteins were subsequently denatured by the addition of 10 mM Tris (2-carboxyethyl) phosphine (TCEP), carbamidomethylated with 40 mM chloroacetamide, and digested with trypsin. The trypsin:protein ratio was 1:100, and the digestion was performed in 100 mM Tris-HCl pH 8.1 buffer. The resulting tryptic peptides were purified and subjected to liquid chromatography coupled to tandem mass spectrometry (LC-MS/MS) on Q Exactive Plus mass spectrometer (Thermo Scientific) as described previously.^92^

#### dsRNA production

DNA templates containing coding sequences for *RpS19a* or *dNep1* were produced by PCR from genomic DNA extracted from w^1118^ flies. Initial PCR amplification (98°C for 2 min, followed by 4 cycles of: 98°C/10 s, 57°C/20 s, 72°C/20 s, 33 cycles of: 98°C/10 s, 66°C/20 s, 72°C/20 s, final extension 72°C/2 min) was further amplified in a 100 μL reaction (98°C for 2 min, followed by 37 cycles of: 98°C/10 s, 66°C/20 s, 72°C/20 s, final extension 72°C/2 min). The PCR product was purified and then used for *in vitro* transcription of dsRNA with a Promega RiboMAX kit. 10 μg template DNA was used per reaction. An agarose gel was used to determine dsRNA size and estimate concentration. Details of primers used can be found in the key resources table.

#### S2 cell transfection

*Drosophila* Schneider’s S2 cells (ThermoFisher R69007) were seeded in 10 cm culture dishes (1 × 10^6^ cells/mL) in Gibco Schneider’s S2 cell medium with 10% FBS, at 28°C. S2 cells were transfected as previously described^93^ using effectene reagent with 7.5 μg of dsRNA for *RpS19a* or *dNep1* for 72 h before collection for polysome profiling.

#### Polysome profiling

Cells were incubated with cycloheximide at a final concentration of 100 μg/mL in growth media for 15 min, cells were pelleted and washed twice with S2 polysome buffer (50 mM Tris, 100 mM NaCl, 5 mM MgCl_2_ containing 10 μg/mL freshly added cycloheximide). Cells were then lysed with S2 cell polysome lysis buffer (50 mM Tris, 100 mM NaCl, 5 mM MgCl_2_, 10 μg/mL cycloheximide, 0.5% Triton X-100, 30 mM DTT, 1× PhosStop, 1× Protease Inhibitors (Thermo Fisher Scientific, cat 87785) and Superase In RNase inhibitor as recommended by manufacturer), volume was adjusted to a final concentration of 8 × 10^6^ cells/100 μL. Lysates were clarified by centrifugation at 16,000×*g* for 15 min at 4°C, the supernatant was removed and stored. 400 μL of the clarified lysate, equivalent to the 3.2 × 10^7^ original cells, was loaded onto a continuous 10–40% sucrose gradients (formed with a 5 mM Tris-HCl pH 7.4, 1.5 mM KCl, 2.5 mM MgCl_2_ buffer) generated using an ISCO gradient maker. Samples were separated by centrifugation using an SW41 rotor spun at 36,000 rpm for 2 h 15 min at 4°C, and fractionated (14 × 1 mL) using a Foxy Jr fraction collector. Absorbance at 260 nm was determined with an ISCO UA-6 absorbance detector.

#### Collection of S2 cell material for mass spectrometry

Analysis of polysome profiles, performed as described above, identified fractions 7–12 as containing polysomes with 2 or more ribosomes associated with mRNA. 250 μL from each of the fractions 7–12 was collected and pooled to extract protein for mass spectrometry, along with 10% of total cell lysate. For the extraction, 1,000 μL of methanol was initially added to each fraction, and after thorough mixing, 250 μL of chloroform was added, samples were then incubated at −20° overnight. Samples were then vortexed at room temperature, and 750 μL of deionised water added, samples were vortexed again and then centrifuged immediately for 5 min at 20,000×*g*. The upper aqueous layer was discarded, and another 1,000 μL of methanol added, samples were inverted 3 times and then centrifuged at 20,000×*g* for 5 min. All liquid was removed and pellet air dried. Protein pellets were then re-suspended and pooled in 20 μL of detergent-free 100 mM NaOH. Once resuspended, 80 μL of 50 mM Tris pH 7.5 was added and samples stored at −20°C. LC-MS/MS was performed as for lymph glands.

#### S2 cell RNA-seq sample collection for differential translation analysis

375 μL of polysome fractions 9–12 containing mRNAs associated with 3 or more ribosomes, thus comprising efficiently translated mRNAs, were pooled and RNA-extracted for RNA-seq, along with 10% of total cell lysate, using a Promega Reliaprep mini extraction kit. RNA was eluted in 30 μL nuclease-free water, RNA quality was checked using an Agilent Bioanalyzer and RNA concentration was measured using Qubit RNA (broad range) reagents. 1 μg of RNA in 50 μL nuclease-free water was used to prepare sequencing libraries. S2 cell samples were sequenced using NextSeq Illumina High output flowcell and 75 bp single-end reads.

#### Ribosome 40S and 60S subunit fractionation

200 μL of lysate, equivalent to 1.6 × 10^7^ cells, were loaded onto continuous 5–30% sucrose gradients (5 mM Tris-HCl pH 7.4, 1.5 mM KCl, 2.5 mM MgCl_2_) generated using an ISCO gradient maker. Samples were separated by centrifugation using an SW41 rotor at 36,000 rpm for 4 h at 4°C.

#### RNA collection and nanopore sequencing

Oxford Nanopore Technologies direct RNA-seq (ONT DRS) for single-molecule long-read direct RNA-seq of individual rRNAs was performed on S2 cell nuclear lysates for dNep1 KD and control, for detection of modified and unmodified nucleotides on 18S rRNA (Ψ/methylation). PBS-washed pelleted cells were briefly lysed in 400 μL non-denaturing buffer (25 mM HEPES-KOH pH 7.6, 50 mM KCl, 5.1 mM MgCl_2_, 2 mM DTT, 0.1 mM EDTA, 5% v/v glycerol, 2 × Protease Inhibitors (Thermo Fisher Scientific), 0.5% Igepal CA-630), and 10 μL of 20 U/μL SUPERase (Invitrogen) immediately added. Cells were disrupted by 5 cycles of pipetting using a low-gauge needle followed by 8 cycles using a high-gauge needle. Nuclei were pelleted by centrifugation at 12,000×*g* for 5 min at 4°C. RNA was then extracted from the nuclear pellet using a PureLink Mini RNA kit (Thermo Fisher Scientific). For unbiased library construction and capture of rRNA intermediates, de-proteinised nuclear RNA fraction was polyadenylated with *E. coli* poly(A) polymerase^94^ prior to the library input. Libraries were constructed using direct RNA-seq kits SQK-RNA002 according to the manufacturer’s recommendations. Sequencing was performed using MinION equipped with FLO-MIN106D R9.4.1 flowcells and generated >1M reads per sample.

### Quantification and statistical analysis

#### Image analysis

Images were processed and prepared using FIJI and Adobe Photoshop CS5/CC. Serial Z stacks were 3D reconstructed in Imaris 9.0.2. Regions of interest were identified using DAPI staining and manually drawing multiple contour lines to create a quantifiable surface. “Spot” analysis was performed for EdU and Hindsight staining to produce a cell count per region of interest, while “Surface” analysis was used for NimC1, caspase, *dome-GFP* and *puc-GFP* analysis to provide a volume of staining per primary lymph gland lobe.

#### Lymph gland RNA-seq bioinformatic analysis

RNA-Seq sequences were aligned to the *Drosophila melanogaster* genome Flybase release 6.10 using Tophat2 v2.1.1 with the default settings. The gene counts were performed using HTSeq v0.6.1 Python package.^95^ Broad separation between control and RpS19a knockdown lymph glands was verified using a Principal Component Analysis (PCA). Differential gene expression analysis was performed on raw gene-level counts with the DESeq2 v1.10.1 R package^42^ to normalise for differences in library size and to remove lowly expressed genes, with a False Discovery Rate (FDR) cutoff 1%. Gene Ontology analysis of Entrez IDs associated with significantly altered genes was performed using the clusterProfiler 2.4.3 R package.^43^ The Benjamini-Hochberg multiple testing correction method was used and adjusted *p*-value cutoff of 0.05 was applied. The clusterProfiler filtering function was applied to exclude parent terms, where applicable.

#### Lymph gland mass spectrometry bioinformatic analysis

The MaxQuant software suite^96^ (LFQ package v1.5.3.30, Andromeda search engine) was used to obtain both protein identifications using an UniProtKB Drosophila database (downloaded July 2016) as a reference and accurate protein quantification measurements. The parameters used were, fixed modification: Carbamidomethylation, variable modification: Oxidation @ M, Acetylation @ Protein N-terminus. The data were normalized based on the assumption that the majority of proteins do not change between the different conditions. Data integration, filtering and statistical analysis was performed in Perseus^97^ v1.5.4.0 after contaminants, “only identified by site” matches and reverse sequences were filtered out. The LFQ data was converted to log_2_ scale, samples were grouped by conditions, and missing values were imputed based on normal distributions after all proteins were eliminated that had 2 or less valid values. Protein fold-changes were calculated, and their significance was determined using a two-sided *t* test with error-corrected *p*-values. An FDR cutoff of 0.05 was applied to determine statistical significance. Figures were generated in R using custom scripts.

#### S2 cell mass spectrometry

Mass spectrometry bioinformatic analysis was performed as for *in vivo* mass spectrometry on lymph glands, using MaxQuant software v1.6.016 and an UniProtKB *Drosophila* database downloaded January 2018 as a reference. Data integration, filtering and statistical analysis was performed in Perseus v1.6.0.7.

#### Analysis of differential translation

Reads from S2 cell samples exhibited sequencing noise detected by FastQC, therefore the first 10 bases were trimmed using Trimmomatic using sliding-window approach. Trimmed reads were aligned to the *Drosophila melanogaster* Ensembl genome BDGP6 using HiSAT2 v2.1.0 with default settings. Gene-level counts were performed using featureCounts Subread 1.6.4. Analysis of differential translation was performed using anota2seq package^54^^,^^55^ with normalize = TRUE and filterZeroGenes = TRUE options enabled. PCA plot was used to verify clustering of samples. The anota2seq “batchVec” option was used to correct for batch effects between technical triplicates. Default value of FDR 0.15 was used to determine statistical significance. Gene Ontology and KEGG pathway analysis of significantly altered genes was performed using clusterProfiler 2.4.3.

#### Ribosome 40S and 60S subunit quantification

Area under curve for 40S and 60S peaks was calculated using GraphPad Prism, by aligning absorbance values for the 60S peak to a single timepoint for all samples and measuring from the beginning of the 40S peak. The baseline was determined individually for each profile by using the lowest absorbance value between 40S and 60S peaks.

#### Nanopore signal processing

Fast5 files from each sample were basecalled using Guppy version 5.0.7 and mapped to *Drosophila melanogaster* pre-rRNA sequence NR_133549.1 using Minimap2 with the parameters “-ax map-ont -k14”. The resulting alignments were filtered with samtools v1.12 such that unmapped reads (-F 4) and non-primary alignments were removed (-F 2324). Mature rRNA reads were isolated using bedtools *intersect* (version 2.28.0) by removing reads that intersect with pre-rRNA intervals (NR_133549.1: 0–861, 2857–4148) with the parameters “-wa -v”. Mature rRNA was further down-sampled to only include 18S rRNA reads in the subsequent Nanocompore analysis. To separate 18S rRNA reads, samtools *view* was used with the coordinates NR_133549.1:862–2856 specified. The separated mature 18S rRNA signals were aligned to the reference using Nanopolish *eventalign* using the options “–scale-events –signal-index –samples –print-read-names”. The Nanopolish output was then collapsed using NanopolishComp *Eventalign_collapse*. dNep1 KD and control collapsed signals were then compared using Nanocompore *SampComp*. The command was run through the Python API with the parameters “max_invalid_kmers_freq 0.6, min_coverage 20, downsample_high_coverage 1000, comparison_methods ‘GMM’, ‘TT’, logit T”.

#### Statistics

All statistical tests that were not part of the RNA-Seq or mass spectrometry analysis were performed with Graphpad Prism 7 using unpaired 2-tailed *t* test with 95% confidence interval. In all figures unless otherwise specified, the error bars represent mean and SD, and significance represented according to the Graphpad classification ∗ (*p* = 0.01–0.05), ∗∗ (*p* = 0.001–0.01), ∗∗∗ (*p* = 0.0001–0.001) and ∗∗∗∗ (*p* < 0.0001). n for confocal image quantification represents lymph glands from individual animals, sampled across at least 3 independent experiments. Differential gene expression significance cutoff was set at False Discovery Rate (FDR) cutoff 1% (*p*_adj_ < 0.01). Mass spectrometry FDR cutoff of 0.05 was applied to determine statistical significance. Differential translation default value of FDR 0.15 was used to determine statistical significance.

## Supplemental information

Document S1. Figures S1–S10

Data S1. Differential gene expression in lymph glands following RpS19a depletion

Data S2. Gene Ontology analysis of differentially upregulated and downregulated genes in lymph glands following RpS19a depletion

Data S3. Mass spectrometry analysis of differential protein abundance in lymph glands following RpS19a depletion

Data S4. Gene Ontology analysis of differentially upregulated and downregulated proteins following RpS19a depletion in lymph glands, identified with mass spectrometry analysis

Data S5. Mass spectrometry analysis of whole cell lysates (inputs) and polysomes for differential protein abundance following RpS19a depletion in S2 cells

Data S6. Analysis of differential translation in S2 cells following RpS19a depletion
