# Supplementary material for: RpS19a depletion alters the hematopoietic translatome to drive tissue overgrowth
Source: iScience. 2026 Sep 15;29(10):117515. doi: 10.1016/j.isci.2026.117515 (PMC13594744; doi:10.1016/j.isci.2026.117515)
Supplement: Supplementary file 1 [file mmc1.pdf]

iScience, Volume *29*

Supplemental information

RpS19a depletion alters the hematopoietic translatome to drive tissue overgrowth

Naomi C. Mitchell, Arjun Chahal, Tanya Javaid, Katrina Woodward, Ralf B. Schittenhelm, Cheng Huang, Julie Lorent, Jiwon Lee, Melanie Rug, Amee J. George, Ross D. Hannan, Ola Larsson, Anthony W. Purcell, Eduardo Eyras, Nikolay Shirokikh, Teresa T. Bonello, Leonie M. Quinn, and Olga Zaytseva

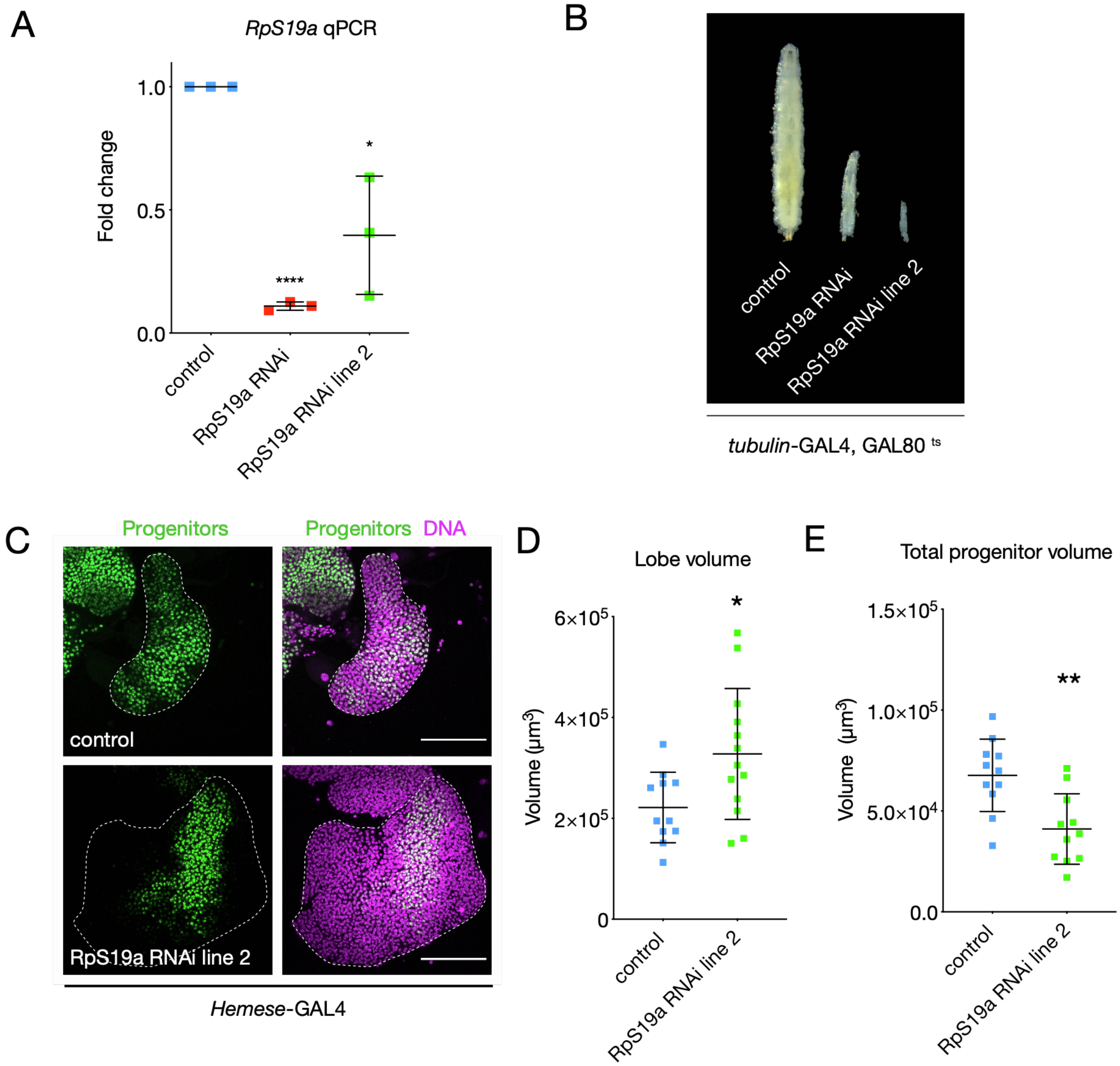

Figure S1. Ubiquitous depletion of RpS19a results in impaired growth, while depletion in the lymph gland results in overgrowth, related to Figure 1

(A) qPCR shows efficiency of KD for RpS19a line 1 (****p<0.0001, Student t-test) and 2 (*p=0.0122, Student t-test). Larvae were raised for 7 days at 18°C and collected 48 hours after shift to 29°C. N=3 biological replicates of tissue pooled from 10 larval heads. (B) Ubiquitous RpS19a KD with either of two independent RNAi constructs using *tubulin*-GAL80^{ts}/+; *tubulin-GAL4*/+ impairs larval growth. (C) Confocal imaging for *Hemese-GAL4*/+ control & *Hemese-GAL4*/RpS19a RNAi 2 lymph glands. 1° lobe outlined. Late 3rd instar larvae were collected 120 hours post egg laying at 29°C. Scale bar is 100 µm. (D) Quantification of 1° lobe volume (p=0.0239, Student t-test, n>11 biological replicates from >3 independent experiment repeats) and (E) total progenitor volume (p=0.0021, Student t-test, n>11 biological replicates from >3 independent experiment repeats). Error bars represent mean and SD.

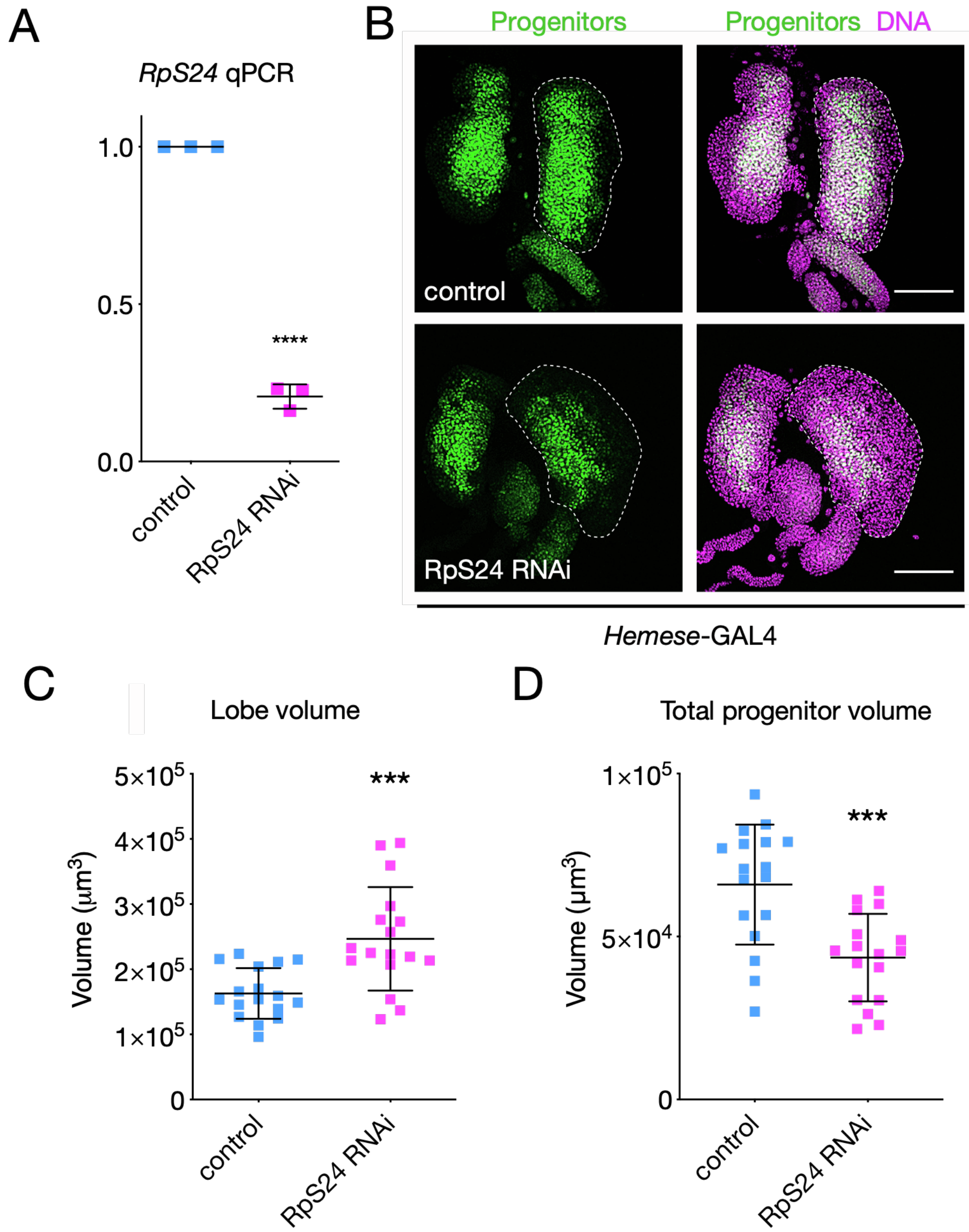

Figure S2. Depletion of RpS24 in the lymph gland results in overgrowth and progenitor loss, related to Figure 1

(A) qPCR shows efficiency of KD for RpS24 RNAi ($p<0.0001$, Student t-test, n=3 biological replicates of tissue pooled from 10 larval heads). Larvae were raised for 7 days at 18°C and collected 48 hours after shift to 29°C. (B) Confocal imaging for *Hemese-GAL4*/+ control & *Hemese-GAL4*/RpS24 RNAi lymph glands. 1° lobe outlined. Late 3rd instar larvae were collected 120 hours post egg laying at 29°C. Scale bar is 100 µm. (C) Quantification of 1° lobe volume ($p=0.0007$, Student t-test, n=17 biological replicates from >3 independent experiment repeats) and (D) total progenitor volume ($p=0.0003$, Student t-test, n=17 biological replicates from >3 independent experiment repeats). Error bars represent mean and SD.

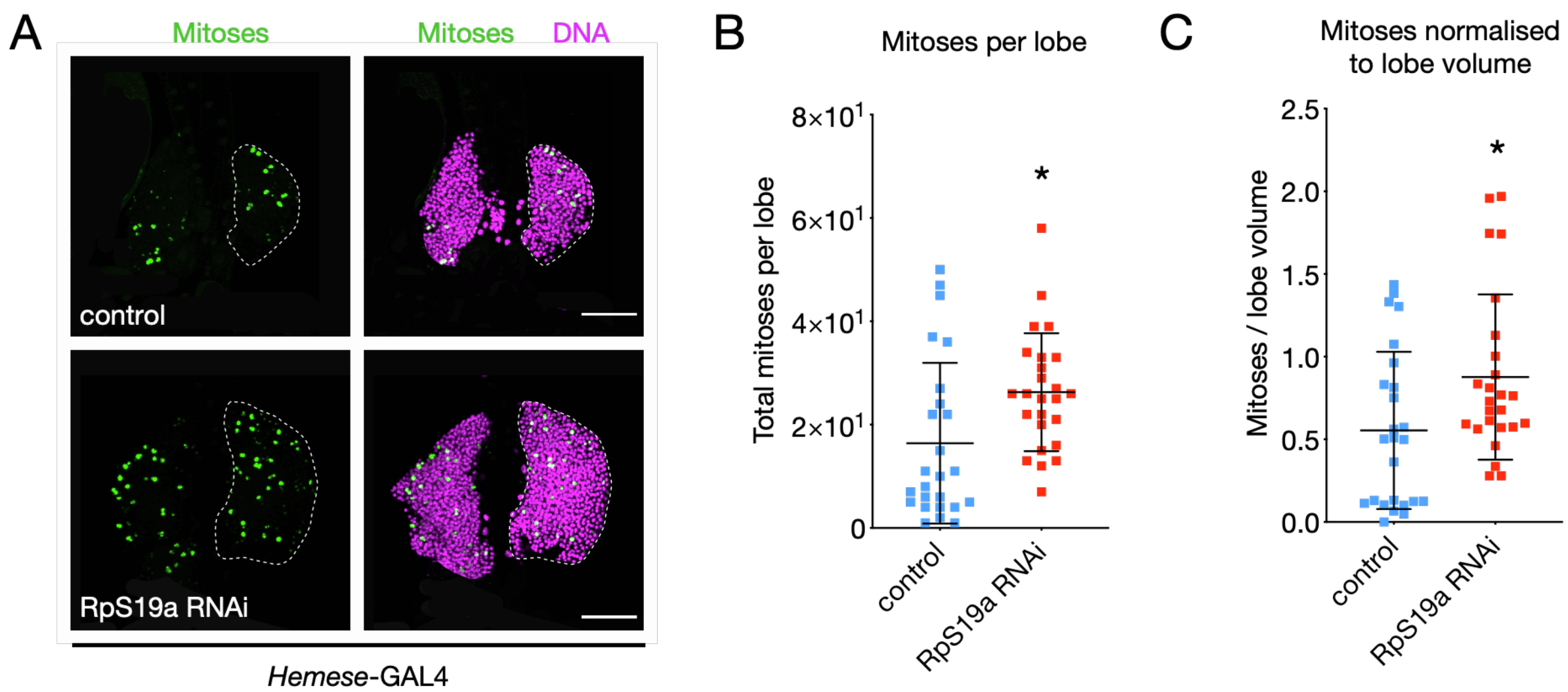

Figure S3. Depletion of RpS19a in the lymph gland results in elevated mitosis, related to Figure 1

(A) Representative images for Hemese-GAL4/+ control and Hemese-GAL4/RpS19a RNAi 1 lymph glands stained with the anti-pH3 antibody; 1° lobe outlined. Late 3rd instar larvae were collected 120 hours post egg laying at 29°C. Scale bar is 100 µm. (B) Quantification of the number of pH3-positive cells per lobe ($p=0.0137$, Student t-test, $n=25$ biological replicates from >3 independent experiment repeats) and (C) pH3-positive cells normalised to lobe volume ($p=0.0235$, Student t-test, $n=25$ biological replicates from >3 independent experiment repeats). Error bars represent mean and SD.

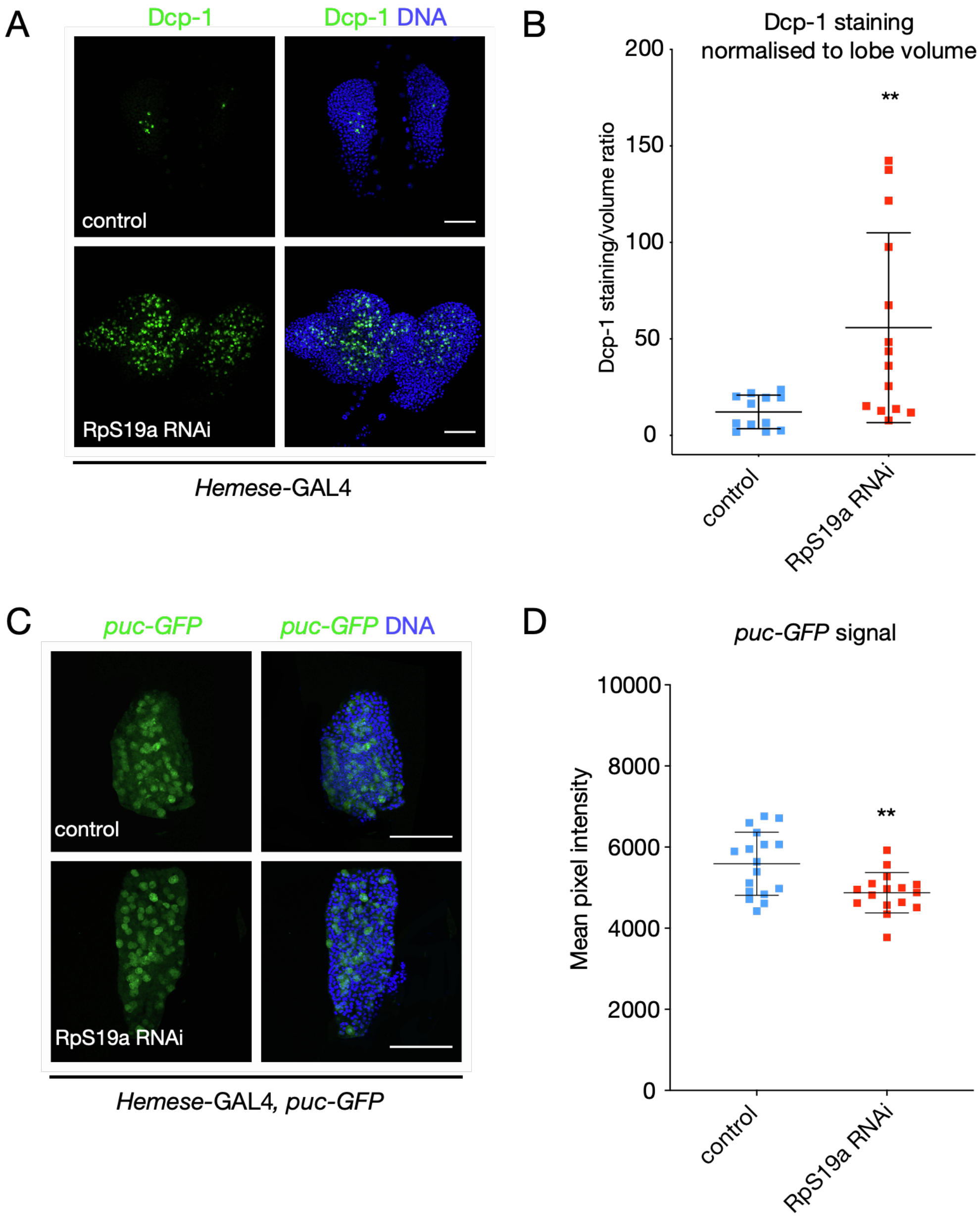

Figure S4. Depletion of RpS19a in the lymph gland results in apoptosis, related to Figure 1

(A) Anti-cleaved caspase 1 (Dcp-1) for control and RpS19a KD (scale bars = 100 µm) and quantification of combined Dcp-1 staining volume as proportion of total volume determined by DAPI ($p = 0.0058$, Student t-test, n>13 biological replicates from >3 independent experiment repeats). (B) *puc-GFP* for representative lymph glands (scale bars = 100 µm) and quantification ($p = 0.0039$, Student t-test, n>16 biological replicates from >3 independent experiment repeats). Late 3rd instar larvae were collected 120 hours post egg laying at 29°C. Error bars represent mean and SD.

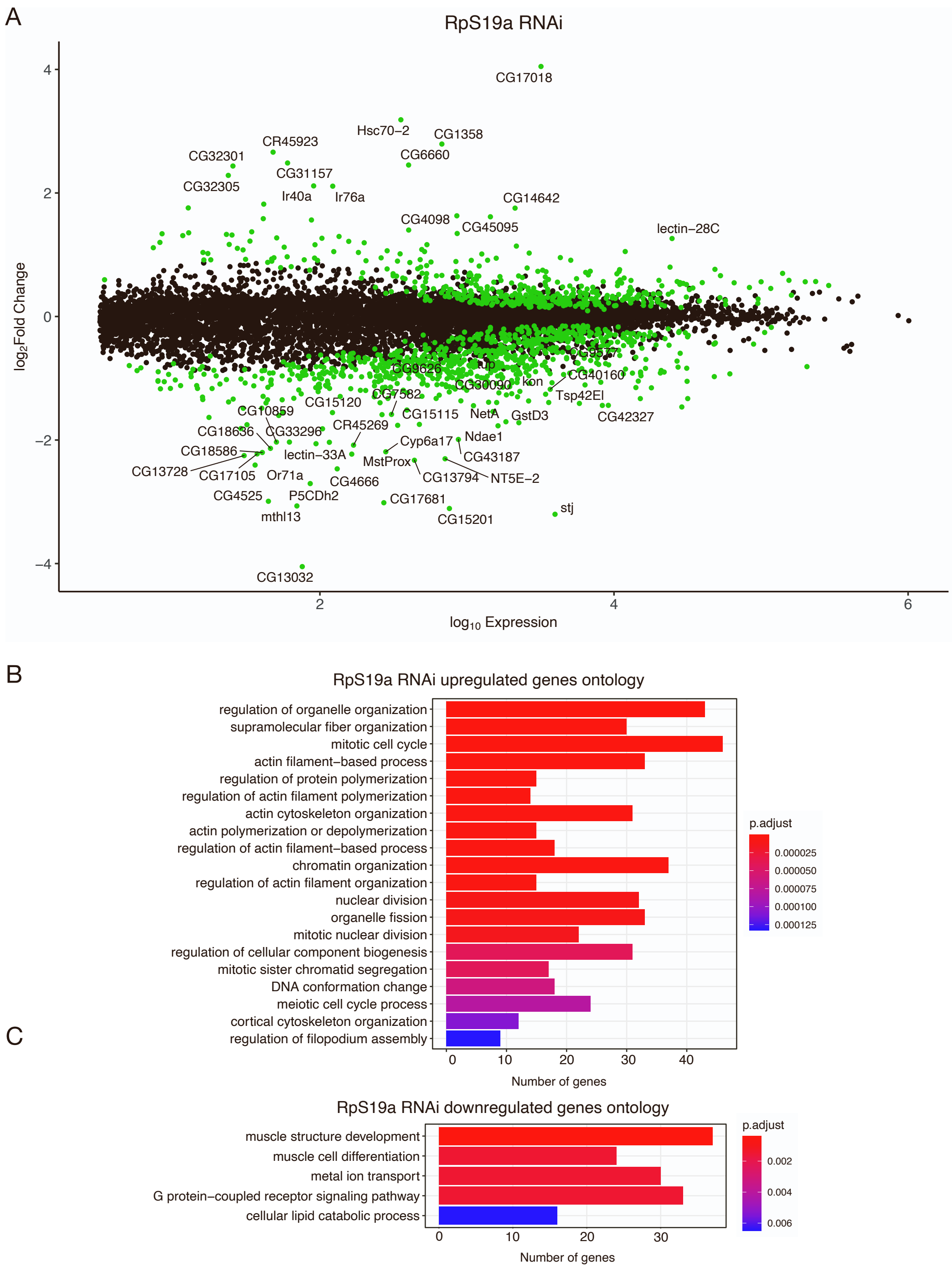

Figure S5. *Hemese-GAL4* driven RNAi knockdown of RpS19a results in differential gene expression in lymph glands, related to Figure 2

(A) DE genes identified by RNA-Seq in RpS19a-depleted lymph glands compared to control. Statistically significant up- or down-regulated genes at FDR < 0.01 are shown in green. N=3 biological replicates of tissue pooled from 40-120 lymph glands. (B) Gene ontology analysis using clusterProfiler for upregulated genes. (C) Gene ontology analysis for downregulated genes. See also Data S1-S2.

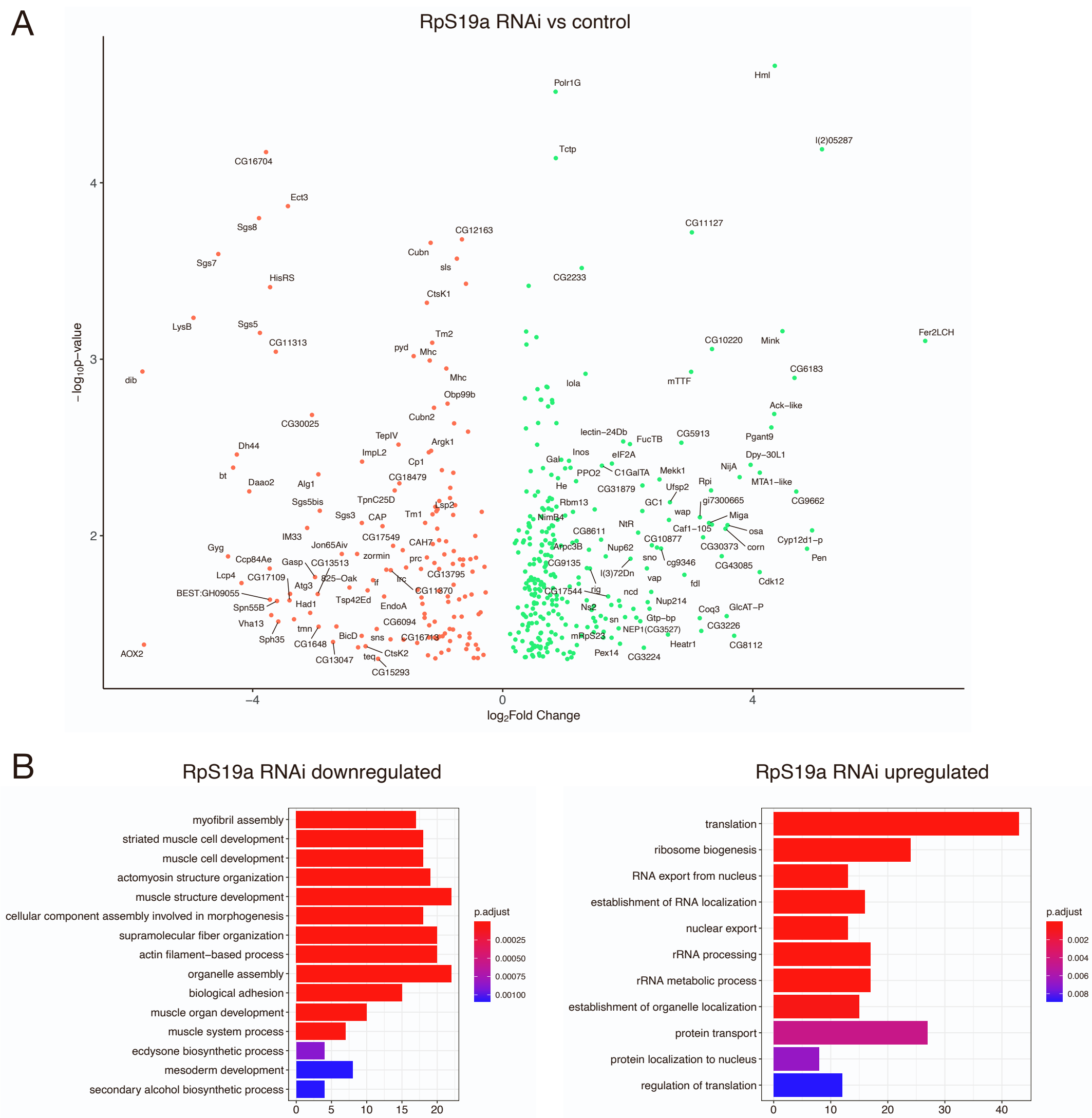

C

Translation initiation

	log2FC
eIF2A	1.75
eIF3b	0.59
eIF3h	0.45
eIF3k	0.69
eIF4A	0.45
eIF4G1	0.52
eIF6	0.37

Translation elongation

	log2FC
eEF1-beta	0.67
eEF1-delta	0.24
eEF2	0.49

40S subunits

	log2FC
RpS4	0.80
RpS12	0.46
RpS15Aa	0.65
RpS18	0.35

60S subunits

	log2FC
RpL7-like	0.50
RpL9	0.48
RpL10Ab	0.25
RpL23	0.51
RpL23A	0.41
RpL24	0.62
RpL30	0.98
RpL31	0.38
RpL35	0.57
RpLP0	0.51

Figure S6. RpS19a KD alters the lymph gland proteome, related to Figure 2

(A) Volcano plot for differentially expressed proteins in RpS19a KD lymph glands compared with control (FDR < 0.05). N=3 biological replicates of tissue pooled from 200-275 lymph glands. (B) Gene ontology classes for differentially expressed proteins. (C) Log_2(fold change) values for differentially expressed translational machinery and RPs (FDR<0.05). See also Data S3-S4.

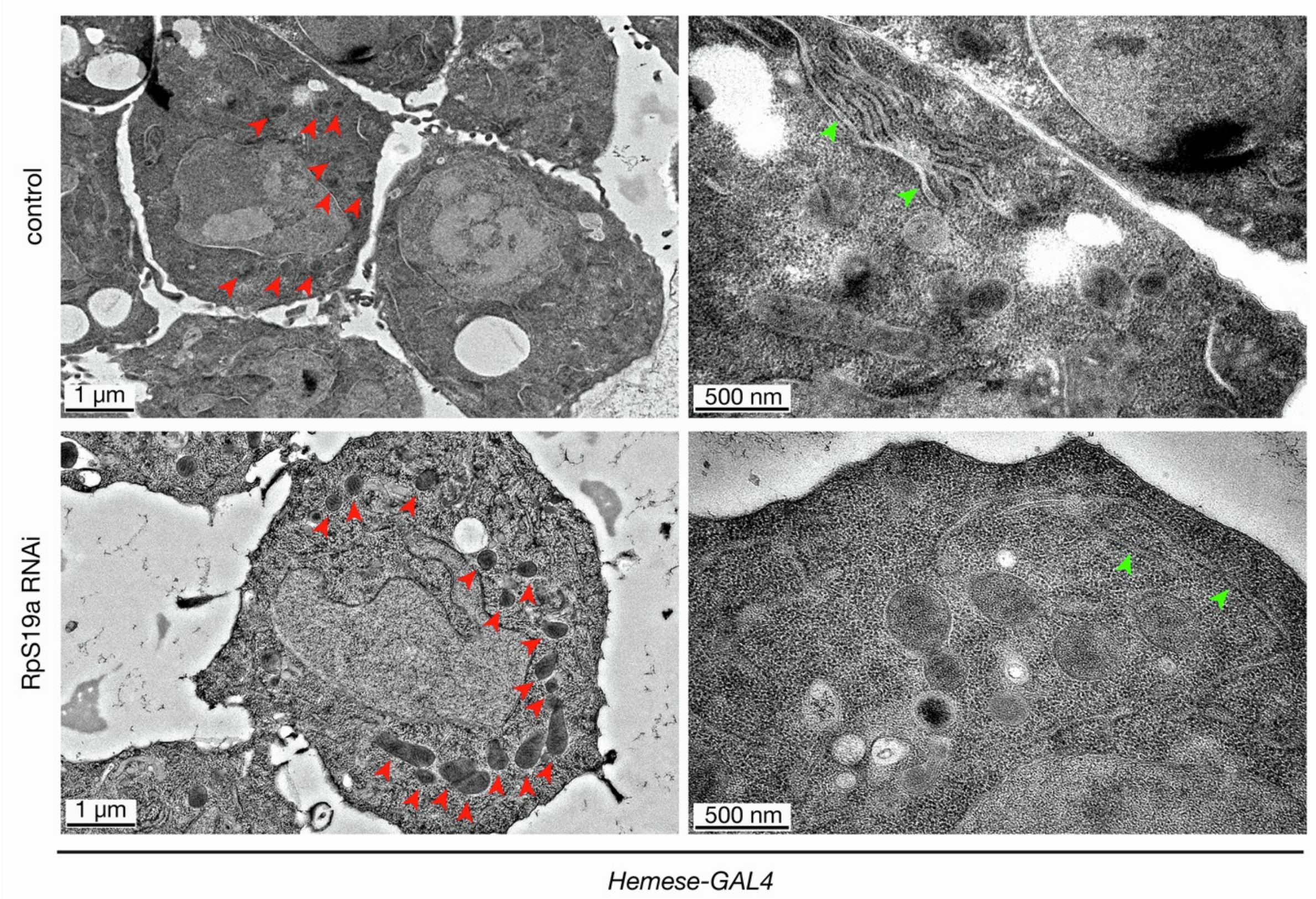

Figure S7. Transmission electron micrographs for control and RpS19a KD lymph gland cells, related to Figure 2

Red arrowheads indicate mitochondria and green arrowheads indicate endoplasmic reticulum with associated ribosomes (black dots). Scale bars = 1 μm (left panels) and 500 nm (right panels).

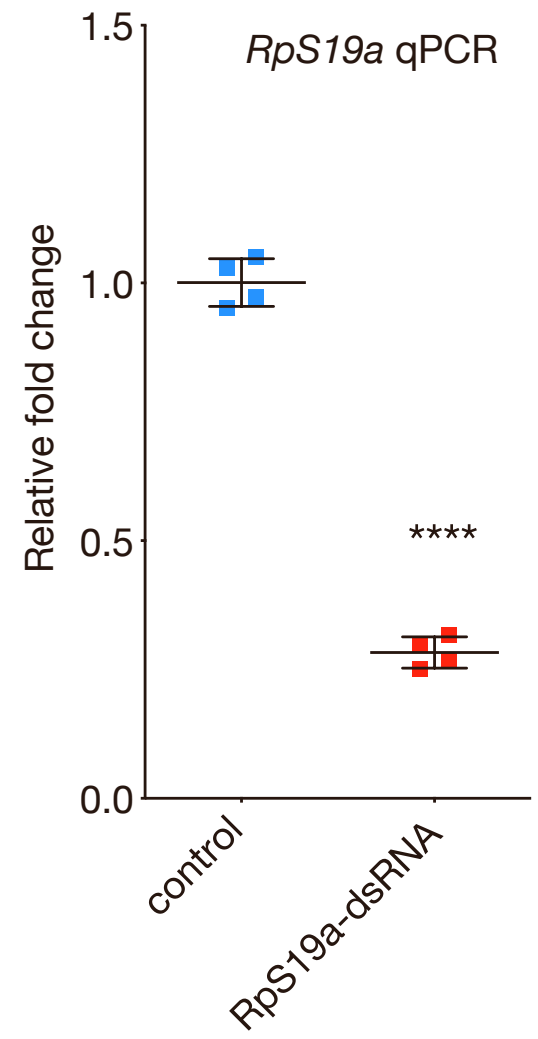

Figure S8. *RpS19a* is depleted by dsRNA in S2 cells, related to Figure 3

qPCR efficiency of KD for *RpS19a* in S2 cells ($p<0.0001$, Student t-test, n=4 biological replicates of tissue pooled from 20 lymph glands), error bars represent mean and SD.

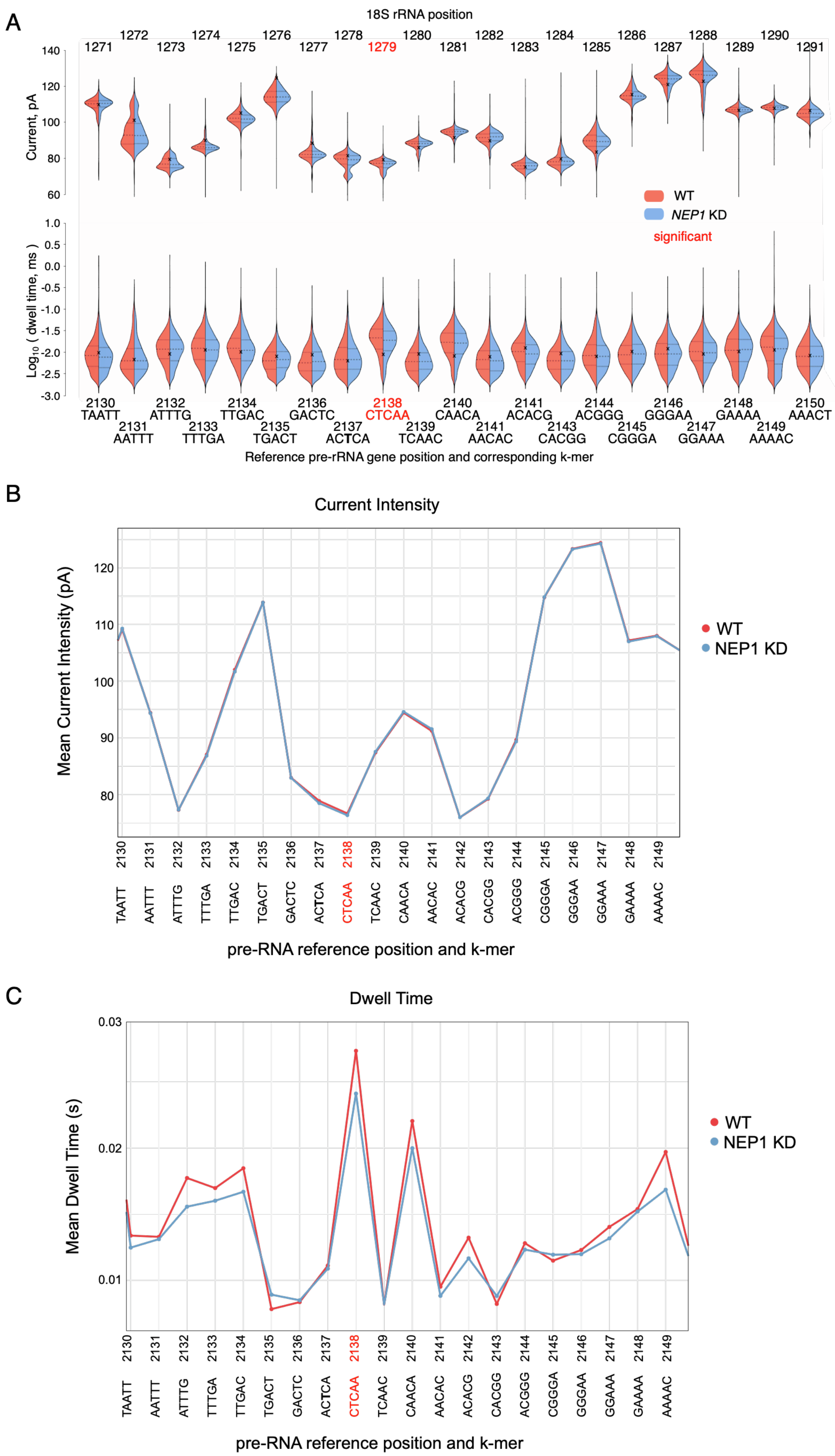

A
18S rRNA position
1271 1272 1273 1274 1275 1276 1277 1278 1279 1280 1281 1282 1283 1284 1285 1286 1287 1288 1289 1290 1291
Current, pA
WT
NEP1 KD
significant
Log10 (dwell time, ms)
2130 TAATT
2131 AATTT
2132 ATTTG
2133 TTTGA
2134 TTGAC
2135 TGACT
2136 GACTC
2137 ACTCA
2138 CTCAA
2139 TCAAC
2140 CAACA
2141 AACAC
2141 ACACG
2143 CACGG
2144 ACGGG
2145 CGGGA
2146 GGGAA
2147 GGAAA
2148 GAAAA
2149 AAAAC
2150 AAACT
Reference pre-rRNA gene position and corresponding k-mer
B
Current Intensity
Mean Current Intensity (pA)
WT
NEP1 KD
pre-RNA reference position and k-mer
C
Dwell Time
Mean Dwell Time (s)
WT
NEP1 KD
pre-RNA reference position and k-mer

Figure S9. Representation of raw direct RNA sequencing Nanopore read data for the region of *Drosophila melanogaster* rRNA covering the consensus dNep1 site, related to Figures 5 and 6
The T of k-mer 5'C**T**CAA3' corresponds to the dNep1 modification site (position 2,138 in pre-rRNA sequence corresponding to position 1,279 in mature 18S rRNA). Positions of the k-mers in the pre-RNA reference gene are indicated (bottom). k-mer showing significant ($p < 0.005$) deviation in the bivariate test and stringent univariate T-tests for intensity and/or dwell time is highlighted (red). (A) Violin plot of signal intensity (top) and dwell time (bottom) distributions per reference k-mer in both WT (red, left halves) and dNep1 KD (blue, right halves) samples across a region of the 18S rRNA. The plot was generated using the Nanocompore Python API. Position of each k-mer in the mature rRNA is indicated (top) and position of the dNep1 consensus modification site highlighted (top; bold font). (B) Mean signal intensity plot for WT (red) and dNep1 KD (blue) samples across pre-rRNA reference region indicated by the cut-in arrows. (C) Same as (B), but for the mean pore dwell time.

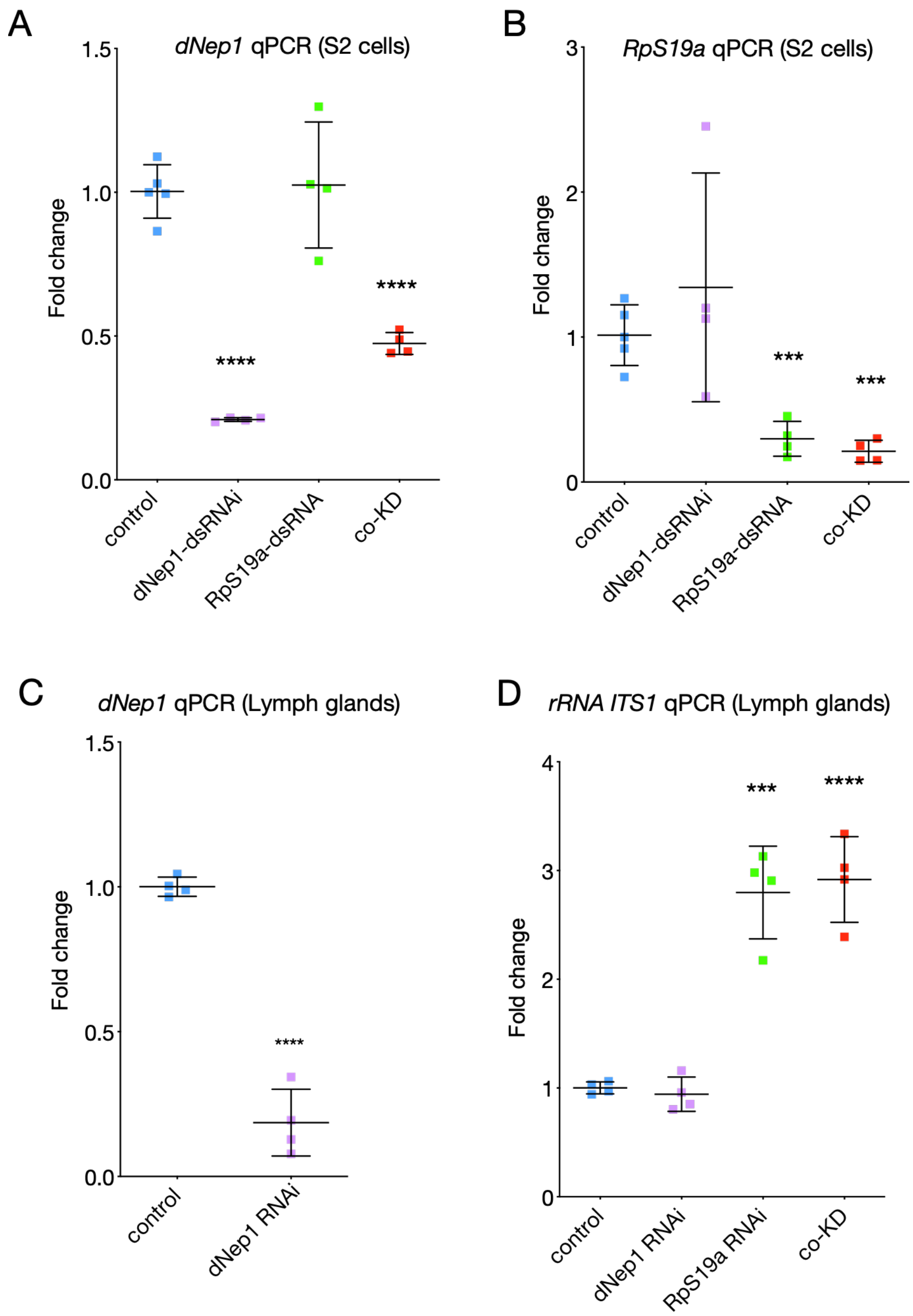

Figure S10. *dNep1* and/or *RpS19a* are depleted by dsRNA in S2 cells and lymph glands, with RpS19a KD resulting in ribosome processing defects, related to Figures 5 and 6. (A) qPCR efficiency of KD for *dNep1* and (B) *RpS19a* in S2 cells. (C) qPCR shows efficiency of KD for *UAS-dNep1* RNAi in lymph glands. (D) rRNA internal transcribed spacer 1 (ITS1) levels in dNep1 and/or RpS19a KD lymph glands. Larvae were raised for 7 days at 18°C and collected 48 hours after shift to 29°C. **** denotes $p<0.0001$, *** $p<0.001$ compared to control using Student t-test, n=4 biological replicates of tissue pooled from 20 lymph glands. Error bars represent mean and SD.
